# The Burden of Osteoarthritis in the Middle East and North Africa Region From 1990 to 2019

**DOI:** 10.3389/fmed.2022.881391

**Published:** 2022-06-23

**Authors:** Ali Shamekh, Mahasti Alizadeh, Seyed Aria Nejadghaderi, Mark J. M. Sullman, Jay S. Kaufman, Gary S. Collins, Ali-Asghar Kolahi, Saeid Safiri

**Affiliations:** ^1^Social Determinants of Health Research Center, Department of Community Medicine, Faculty of Medicine, Tabriz University of Medical Sciences, Tabriz, Iran; ^2^Research Center for Integrative Medicine in Aging, Aging Research Institute, Tabriz University of Medical Sciences, Tabriz, Iran; ^3^Systematic Review and Meta-Analysis Expert Group (SRMEG), Universal Scientific Education and Research Network (USERN), Tehran, Iran; ^4^Department of Life and Health Sciences, University of Nicosia, Nicosia, Cyprus; ^5^Department of Social Sciences, University of Nicosia, Nicosia, Cyprus; ^6^Department of Epidemiology, Biostatistics and Occupational Health, Faculty of Medicine, McGill University, Montreal, QC, Canada; ^7^Centre for Statistics in Medicine, Nuffield Department of Orthopaedics, Rheumatology and Musculoskeletal Sciences, Botnar Research Centre, University of Oxford, Oxford, United Kingdom; ^8^NIHR Oxford Biomedical Research Centre, Oxford University Hospitals NHS Foundation Trust, Oxford, United Kingdom; ^9^Social Determinants of Health Research Center, Shahid Beheshti University of Medical Sciences, Tehran, Iran; ^10^Connective Tissue Diseases Research Center, Tabriz University of Medical Sciences, Tabriz, Iran

**Keywords:** osteoarthritis, epidemiology, prevalence, incidence, burden, Eastern Mediterranean

## Abstract

**Objective:**

We aimed to report the most current data on the prevalence, incidence, and years lived with disability (YLDs) associated with osteoarthritis (OA) for the 21 countries and territories located in the Middle East and North Africa (MENA) region from 1990 to 2019 by age, sex, cause, and sociodemographic index (SDI).

**Methods:**

Publicly available data from the Global Burden of Disease 2019 study were used to report the OA-related burden. Estimates are reported as counts and age-standardized rates, along with their corresponding 95% uncertainty intervals (UIs).

**Results:**

In 2019, the age-standardized prevalence of OA in MENA was 5,342.8 per 100,000 (95% UI: 4,815.9–5,907.8), which is 9.3% higher than in 1990 (8.1–10.5%). Similarly, the age-standardized annual incidence of OA per 100,000 was 430.4 (382.2–481.9), demonstrating a 9.4% increase since 1990 (8.3–10.5). OA was the cause of 185.4 (92.8–370.2) age-standardized YLDs per 100,000 in 2019, which was 10% higher than in 1990 (8.7–11.4). Saudi Arabia, Kuwait, and Iran had the highest OA burden in MENA, while Yemen, Afghanistan, and Sudan had the lowest burden. In all MENA countries, OA affected more women than men, had an increasing burden with increased age, and had the highest impact on the knee, hip, and hand joints, respectively. OA was also positively associated with the SDI.

**Conclusion:**

The burden of OA increased over 1990–2019 in the MENA region. The study emphasizes the importance of early preventative approaches in order to control any future health, economic, and quality of life crises imposed by OA in this region.

## Introduction

Osteoarthritis (OA), which is one of the most common rheumatologic diseases and the most prevalent form of arthritis, is a substantial cause of disability in older adults (1). This disease is associated with the loss and impairment of joint facet cartilage and the inflammation of the synovial spaces (2). In the advanced stages, patients require joint replacement therapy or other invasive procedures (3–5). Different biological and biomechanical risk factors have been investigated, including genetic susceptibility, aging, and excess body weight (6).

OA imposes a devastating economic burden on both the patients and the healthcare system (1, 7), with OA-related costs in the United States estimated to have reached almost 200 billion dollars a year (8). A study in different subgroups of OA patients in United Arab Emirates showed that the annual healthcare cost for each patient was about 350 dollars (9). In the “hard to treat” subgroup, which were contradicted for the conventional non-steroid anti-inflammatory drugs (NSAIDs), the medication costs and procedure costs were 1,813 and 3,005 dollars annually for each patient (9). Furthermore, the burden of OA rises as the population ages (6, 10). It has been estimated that OA accounts for around 8% of the total burden of diseases worldwide, and is more common among women (1, 11, 12). Previous research has shown that globally, the joints most commonly affected by OA are the knee, hip, and hand, with larger joints (e.g., hip and knee) causing more disability (10, 13, 14). The failure of the knee or hip joints may result in the need for joint replacement surgery, if available, which accounts for a substantial proportion of the direct health care costs associated with arthritis (15).

The Global Burden of Disease (GBD) 2017 study reported the prevalence, incidence, and years lived with disability (YLD), due to OA, at the global, regional, and national levels (10). However, estimates for important joints such as OA in the hands were not reported (10). Furthermore, there have been no recent reports focusing on the Middle East and North Africa (MENA) region. The most recent research used data from the GBD 2013 project (16) to report the overall burden of musculoskeletal disorders in the MENA region. The previous study did not include OA-specific data and is also outdated. The present study was conducted to provide the most current data on the prevalence, incidence, and YLDs associated with OA for the 21 countries and territories located in the MENA region from 1990 to 2019 by age, sex, cause, and sociodemographic index (SDI).

## Methods

GBD 2019, the latest iteration of the GBD project, estimated the burden of 369 diseases, from 1990 to 2019, for 204 countries and territories, seven super-regions and 21 regions. A summary of the GBD 2019 methodology, along with the improvements made since GBD 2017, can be found elsewhere (15). Fatal and non-fatal estimates can be obtained from https://vizhub.healthdata.org/gbd-compare/ and http://ghdx.healthdata.org/gbd-results-tool.

The study was approved by the ethics committee of Tabriz University of Medical Sciences. All methods were performed in accordance with the national guidelines and regulations.

### Case Definition and Data Inputs

The Institute for Health Metrics and Evaluation (IHME) reviewed the OA estimates for the hip, knee, hand, and other sites for GBD 2019. Although OA of the spine is relatively common, the symptoms and disability related to the cervical and/or lumbar spine would already be encapsulated within the lower back and neck pain surveillance estimates (15).

The reference case definition was symptomatic OA radiologically confirmed Kellgren-Lawrence grade 2–4. Previous iterations of the GBD project only estimated OA of the hip and knee, but GBD 2019 added OA of the hand and OA in other joints. OA of the hand included any single hand joint, while OA in other joints included any joint other than those of the hand, hip, knee, or spine. Grade 2 symptomatic OA requires one defined osteophyte in the affected joint, as well as pain for at least one of the last 12 months. Grade 3–4 symptomatic OA involves osteophytes and a narrowing of the joint space in the affected joint, while grade 4 requires joint deformity and pain for at least 1 month out of the last 12. The International Classification of Diseases (ICD) 9 code for OA is 715, without individual codes for the different sites, whereas ICD-10 has codes for OA of the hip (M16), knee (M17), trapeziometacarpal joint (M18), and OA in other joints (M19) (15). Although ICD-10 provides more accurate data on the classification of OA, compared to the previous version, it only encompasses the first carpometacarpal joint. In the GBD 2019 study, hand OA refers not only to the trapeziometacarpal joint, but also to any single hand joint, as well as multiple affected joints and generalized hand OA.

The most recent systematic review for hip and knee OA was conducted in GBD 2017. The systematic review of the prevalence, incidence, and mortality used the following databases: MEDLINE, EMBASE, CINAHL, CAB Abstracts, WHO Library (WHOLIS) and OpenSIGLE. The search terms used to investigate the prevalence and incidence could be found elsewhere (15). The results of the previous systematic review were re-viewed in 2019 to add information about the prevalence and incidence of hand OA or OA in other joints. The additional information on the number of studies used in the modeling of OA can be found using the *GBD Data Input Sources Tool* (http://ghdx.healthdata.org/gbd-2019/data-input-sources) (15).

As in previous GBD iterations, hospital inpatient data were not included. The reason for this is that it would not be representative of the true prevalence, and the variation between countries in the proportion of true prevalent cases captured by hospital inpatient data would be likely to vary more than can be adjusted for in a single crosswalk using DisMod-MR 2.1. However, claims data were included from Taiwan (in 2016) and the USA (by state) for 2000, as well as 2010–2016. The review and systematic review identified very few sources for OA in other sites with minimal overlap in the reported site. Therefore, the only data for this model came from the US claims data. Finally, there were some differences in the number of countries with data on hip OA, knee OA, hand OA and OA in other joints; and the additional information are available from: http://ghdx.healthdata.org/gbd-2019/data-input-sources (15).

### Data Processing and Disease Model

Prevalence estimates were calculated by age and sex, where possible. First, if studies reported the prevalence for broad age groups by sex (e.g., prevalence in 15–65-year-old males and females separately), and also by specific age groups, but for both sexes combined (e.g., prevalence in 15–30 year olds, then in 31–65 year olds, for males and females combined), age-specific estimates were split by sex using the reported sex ratio and bounds of uncertainty. Secondly, data which reported the prevalence of OA for both sexes, which could not be split using a within-study ratio, used Meta-Regresssion using the Bayesian, Regularized, Trimmed method (MR-BRT) to separate the data using a sex ratio derived from a meta-analysis of the existing sex-specific data for each type of OA. The female to male ratio was 1.10 (1.09–1.12) for the hip, 1.44 (1.43–1.45) for the knee, and 2.36 (2.33–2.38) for the hand. For OA in other joints, no data reported both sexes together. Where research reported estimates on OA hip and OA knee across age groups spanning 25 years or more, these were split into 5-year age groups, for each type of OA, using the prevalence age pattern estimated by DisMod-MR 2.1, a Bayesian meta-regression tool. The remaining wide age bin data for OA hand were split into 5-year age groups, using the prevalence age pattern of the USA claims data. There were no wide age bin data for OA in other joints (15). The alternative case definitions for hip OA, knee OA and hand OA were accommodated and adjusted using MR-BRT (15).

In the DisMod model, remission was set to 0, as was excess mortality, and it was assumed that there were no cases of OA before the age of 30. However, a remission rate of 0.05 was used for knee OA, to take into account knee replacement therapy. The different models, using country-level covariates, were run to estimate the incidence and prevalence of hip, knee, hand and OA in other sites (15).

### Compilation of Results

Supplementary Table S1 presents the OA severity level, lay description, and disability weights (DWs). The years lived with disability (YLDs) was calculated by multiplying the prevalence in each severity category with the severity-specific DWs. There were no deaths or years of life lost (YLLs) due to OA, and so the DALYs were the same as the YLDs. Additional details about the calculation of YLDs can be found elsewhere (15). Uncertainty was propagated by combining uncertainty from multiple sources, including input data, corrections for measurement error and estimates of residual non-sampling error and by sampling 1000 draws at each computational step. The uncertainty intervals (UIs) were specified to be the 2.5th and 97.5th values of the ordered draws. Smoothing splines models were used to investigate the relationship SDI has with the OA burden for each country in the MENA region (16). The SDI scores range from 0 (less developed) to 1 (most developed), and contains the smoothed gross domestic product per capita (over the previous decade), average years of schooling for the population older than 15 years old, and the total fertility rate for those under the age of 25. The age-standardized point prevalence, incidence and YLD rates were mapped using R software (V 3.5.2).

## Results

### Middle East and North Africa (MENA) Region

There were 24.6 million cases (95% UI 22.0–27.3 million) of OA in the Middle East and North Africa (MENA) region, with an age-standardized point prevalence of 5,342.8 per 100,000 (95% UI 4,815.9–5,907.8), which was 9.3% higher than in 1990 (95% UI 8.1–10.5%). Similarly, the percentage change in the age-standardized rate (ASR) of incidence of OA increased by 9.4% from 1990 to 2019 (95% UI 8.3–10.5), with ~2.3 million cases (95% UI 2.0 to 2.6 million) of OA in 2019, and an ASR of 430.4 (95% UI 382.2–481.9) per 100,000. In the MENA region, OA caused more than 852,000 YLDs (95% UI 425,000–1.6 million), with an ASR of 185.4 per 100,000 (92.8–370.2). The ASR increased by 10% from 1990 to 2019 (95% UI 8.7–11.4) (Table 1).

**Table 1:** Prevalent cases, incident cases and YLDs due to osteoarthritis in the Middle East and North Africa region in 2019 and percentage change of age-standardized rates during 1990–2019 (generated from data available from http://ghdx.healthdata.org/gbd-results-tool).

	**Prevalence (95% UI)**			**Incidence (95% UI)**			**YLDs (95% UI)**		
	**Counts (2019)**	**ASR (2019)**	**Pcs in ASRs 1990–2019**	**Counts (2019)**	**ASRs (2019)**	**Pcs in ASRs 1990-2019**	**Counts (2019)**	**ASRs (2019)**	**Pcs in ASRs 1990–2019**
North Africa and Middle East	24,604,611 (22,080,960, 27,327,135)	5,342.8 (4,815.9, 5,907.8)	9.3 (8.1, 10.5)	2,292,214 (2,035,506, 2,570,668)	430.4 (382.2, 481.9)	9.4 (8.3, 10.5)	852,891 (425,290, 1,687,138)	185.4 (92.8, 370.2)	10 (8.7, 11.4)
Afghanistan	665,279 (590,218, 749,421)	4,874.1 (4,363.9, 5,431.5)	8.5 (5.5, 11.9)	69,705 (60,653, 79,550)	391.1 (346.1, 441.8)	7.8 (4.7, 11.5)	22,313 (11,329, 44,226)	163.9 (83.1, 326.4)	8.5 (4.9, 12.4)
Algeria	1,882,093 (1,681,187, 2,089,085)	5,320.3 (4,765.6, 5,873.4)	10.1 (7, 13.4)	169,449 (149,048, 190,369)	427.5 (378, 478.6)	10 (6.9, 13.6)	65,452 (32,760, 129,991)	185.1 (92.9, 369.4)	10.8 (6.9, 14.5)
Bahrain	72,697 (64,518, 81,706)	5,471.3 (4,915, 6,032.6)	6.7 (3.8, 10)	7,927 (6,928, 8,953)	434.4 (384.2, 483.1)	6.8 (3.7, 10.1)	2,505 (1,248, 4,957)	188.7 (95, 375.5)	6.8 (3.3, 10.8)
Egypt	3,656,548 (3,277,333, 4,064,564)	5,374.1 (4,844.3, 5,936.9)	7.5 (4.3, 11.1)	340,162 (300,085, 381,427)	432.8 (383.2, 484.5)	8 (4.4, 11.5)	127,344 (62,958, 254,833)	187.2 (93.6, 377.1)	7.9 (4.3, 12.3)
Iran (Islamic Republic of)	4,282,092 (3,844,615, 4,781,751)	5,588.2 (5,041.2, 6,228.6)	7 (6.1, 8)	385,983 (342,697, 434,999)	445 (395.9, 501.9)	7.3 (6.4, 8.4)	149,354 (75,490, 293,270)	195.3 (98.7, 381)	7.9 (6.9, 9.1)
Iraq	1,308,928 (1,167,311, 1,459,554)	5,321.5 (4,762.8, 5,895.9)	4.3 (1.2, 7.5)	125,093 (109,828, 141,426)	425.8 (378.8, 475.4)	4.4 (1.2, 7.8)	45,113 (22,709, 89,017)	183.8 (92.7, 367.3)	5.4 (1.7, 9.3)
Jordan	399,351 (357,424, 447,816)	5,470.4 (4,922.8, 6,035.8)	9.6 (6.5, 13)	38,919 (34,279, 43,848)	437.7 (389.5, 489.4)	9.6 (6.1, 13)	13,920 (6,964, 27,536)	191.3 (95.2, 383.9)	10.7 (7.1, 14.5)
Kuwait	183,772 (163,298, 206,192)	5,627.6 (5,049.7, 6,251.9)	9.2 (6.1, 12.6)	20,581 (18,103, 23,375)	451.6 (400.4, 505.7)	10.1 (6.7, 13.9)	6,374 (3,165, 12,712)	196.7 (98.5, 392)	9.5 (5.7, 13.7)
Lebanon	277,812 (250,318, 307,359)	5,370.7 (4,832.9, 5,940.6)	10.6 (7.4, 13.9)	22,535 (19,987, 25,317)	430 (381.5, 483.4)	10.6 (7.3, 14.2)	9,609 (4,871, 18,962)	185.7 (94.2, 366.5)	11 (7.4, 14.9)
Libya	300,298 (268,933, 337,344)	5,416.8 (4,883.3, 6,035.7)	6.9 (3.7, 10.2)	29,168 (25,689, 32,984)	433.9 (385.1, 488.1)	7.3 (3.7, 10.5)	10,363 (5,240, 20,678)	187.6 (94.4, 374.5)	6.8 (3.1, 10.8)
Morocco	1,713,120 (1,533,283, 1,916,511)	5,209.1 (4,679.8, 5,786.7)	9.1 (5.9, 12.4)	151,245 (133,506, 171,160)	418.4 (372.5, 471.3)	9.1 (5.8, 12.4)	59,341 (29,857, 117,164)	180.5 (90.4, 357.2)	9.5 (5.7, 13.5)
Oman	117,159 (104,084, 131,554)	5,361.9 (4,819, 5,945.5)	14.6 (11.3, 18.1)	13,756 (12,170, 15,528)	424.6 (377.4, 475.1)	14.4 (10.9, 18.2)	4,051 (2,027, 8,146)	185.8 (93, 364.3)	15.9 (11.9, 20.1)
Palestine	133,600 (120,025, 149,034)	5,191.1 (4,676, 5,749.6)	4.6 (1.5, 7.7)	12,852 (11,290, 14,494)	415.3 (370.3, 465.4)	4.3 (1, 7.6)	4,590 (2,321, 9,133)	178.7 (89.5, 350.3)	4.5 (1, 8.4)
Qatar	83,742 (73,719, 94,909)	5,508 (4,952.1, 6,115.1)	4.8 (1, 8.1)	10,579 (9,244, 120,09)	439.1 (390.5, 489.9)	6.1 (2.3, 10)	2,878 (1,436, 5,806)	189.5 (95.4, 379.6)	4.4 (0.3, 8.2)
Saudi Arabia	1,500,332 (1,324,931, 1,700,792)	6,601.3 (5,941.5, 7,358.3)	9.7 (7, 12.5)	168,749 (147,967, 193,393)	504 (447.9, 565.1)	11.2 (8, 14.5)	52,545 (26,610, 104,734)	233.1 (118.8, 457.8)	10.7 (7.3, 13.8)
Sudan	1,005,517 (896,511, 1,127,167)	5,081.9 (4,555.5, 5,652.7)	12.5 (8.9, 15.9)	97,698 (86,070, 110,443)	410.3 (363.9, 462.8)	12.7 (9.1, 16.4)	34,719 (17,492, 68,873)	175.8 (88.9, 351.2)	13.4 (9.4, 17.6)
Syrian Arab Republic	698,433 (622,281, 780,321)	5,218.7 (4,677.5, 5,794.9)	6.6 (3.4, 9.9)	62,346 (54,723, 70,683)	420.5 (373.8, 472.9)	6.8 (3.7, 10.2)	24,154 (12,077, 47,650)	180.4 (90.7, 357.1)	6.5 (2.7, 10.1)
Tunisia	684,103 (610,552, 759,467)	5,268.9 (4,723.7, 5,828.4)	8.7 (5.6, 11.7)	57,298 (50,683, 64,574)	422.8 (375.9, 475.5)	8.5 (5, 11.8)	23,773 (12,000, 47,643)	183 (92.6, 368.2)	9 (5.1, 12.7)
Turkey	4,606,241 (4,093,835, 5,152,806)	5,089.5 (4,532.3, 5,694.9)	10.3 (6.7, 14.4)	393,382 (347,298, 440,721)	416.6 (369.7, 466)	9.7 (5.9, 13.4)	159,045 (78,292, 319,394)	175.7 (86.7, 352.9)	11.1 (6.9, 15.9)
United Arab Emirates	339,146 (296,726, 386,821)	5,318.6 (4,780.7, 5,879.2)	8.8 (5.2, 12.4)	46,556 (40,342, 53,570)	423 (377, 477.6)	9.5 (5.7, 12.9)	11,637 (5,755, 23,432)	183.5 (92.6, 362.8)	9.1 (5, 13.1)
Yemen	669,350 (599,689, 748,587)	4,770.5 (4,293.7, 5,293.9)	6.5 (3.3, 10)	65,904 (58,348, 74,424)	383.5 (340.9, 430.6)	7.3 (3.9, 10.5)	22,943 (11,442, 45,262)	163.7 (82.1, 326)	7 (3.3, 11)

### National Level

In 2019, the number of prevalent cases of OA in the MENA countries ranged from 72,697 to 4.6 million, with the ASR prevalence ranging from 4,770.5 to 6,601.3 per 100,000 population. Saudi Arabia [6,601.3 (95% UI 5,941.5–7,358.3)], Kuwait [5,627.6 (95% UI 5,049.7–6,251.9)], and Iran [5,588.2 (95% UI 5,041.2–6,228.6)] had the three highest age-standardized prevalence of OA in the MENA region. In contrast, the countries with the lowest rates in 2019 were Yemen [4,770.5 (95% UI 4,293.7–5,293.9)], Afghanistan [4,874.1 (95% UI 4,363.9–5,431.5)], and Sudan [5,081.9 (95% UI 4555.5–5652.7)] (Supplementary Table S1). The countries with the highest estimated percentage change in the age-standardized prevalence of OA over the period 1990–2019 were Oman [14.6% (95% UI 11.3–18.1%)], Sudan [12.5% (95% UI 8.9–15.9%)], and Lebanon [10.6% (95% UI 7.4–13.9%)]. In contrast, Iraq [4.3% (95% UI 1.2–7.5%)], Palestine [4.6% (95% UI 1.5–7.7%)], and Qatar [4.8% (95% UI 1–8.1%)] had the lowest changes in the age-standardized prevalence (Supplementary Table S1). The age-standardized prevalence of OA and their changes during the study period (1990–2019) are presented in Supplementary Figures S1, S2, respectively.

In 2019, the number of incident cases of OA in the MENA region ranged from 7,927 to 393,382, while the ASR ranged from 383.5 to 504 per 100,000 population. Saudi Arabia [504 (95% UI 447.9–565.1)], Kuwait [451.6 (95% UI 400.4–505.7)], and Iran [445 (95% UI 395.5–501.9)] had the highest age-standardized incidence in 2019. In contrast, Yemen [383.5 (95% UI 340.9–430.6)], Afghanistan [391.1 (95% UI 346.1–441.8)], and Sudan [410.3 (95% UI 363.9–462.8)] had the lowest ASRs in 2019 (Supplementary Table S2). Furthermore, the age-standardized incidence of OA increased in all MENA countries over the measurement period, with Oman [14.4% (95% UI 10.9–18.2%)], Sudan [12.7% (95% UI 9.1–16.4%)], and Saudi Arabia [11.2% (95% UI 8.0–14.5%)] having the highest increases. In contrast, the lowest increases were estimated to be in Palestine [4.3% (95% UI 1.0–7.6%)], Iraq [4.4% (95% UI 1.2–7.8%)], and Qatar [6.1% (95% UI 2.3–10%)] (Supplementary Table S2). The ASRs of OA, and their changes during the period 1990–2019, are presented in Supplementary Figures S3, S4, respectively.

The YLDs due to OA in 2019 ranged from 2,505 to 159,045, while the age-standardized YLDs were between 163.7 and 233.1 per 100,000 population. The age-standardized YLD rates showed a similar pattern to the age-standardized incidence rates, with Saudi Arabia [233.1 (95% UI 118.8–457.8)], Kuwait [196.7 (95% UI 98.5–392.0)], and Iran [195.3 (95% UI 98.7–381.0)] having the highest rates. In contrast, Yemen [163.7 (95% UI 82.1–326.0)], Afghanistan [163.9 (83.1–326.4)], and Turkey [175.7 (95% UI 86.7–352.9)] had the lowest rates (Supplementary Table S3). In addition, the changes in the age-standardized YLDs were highest in Oman [15.9% (95% UI 11.9–20.1%)], Sudan [13.4% (95% UI 9.4–17.6%)], and Turkey [11.1% (95% UI 6.9–15.9%)]. In contrast, the lowest increases were seen in Qatar [4.4% (95% UI 0.3–8.2%)], Palestine [4.5% (95% UI 1.0–8.4%)], and Iraq [5.4% (95% UI 1.7–9.3%)] (Supplementary Table S3). The ASR YLDs and their changes during the period 1990–2019 are presented in Supplementary Figures S5, S6, respectively.

### Age and Sex Patterns

**Figure 1:**
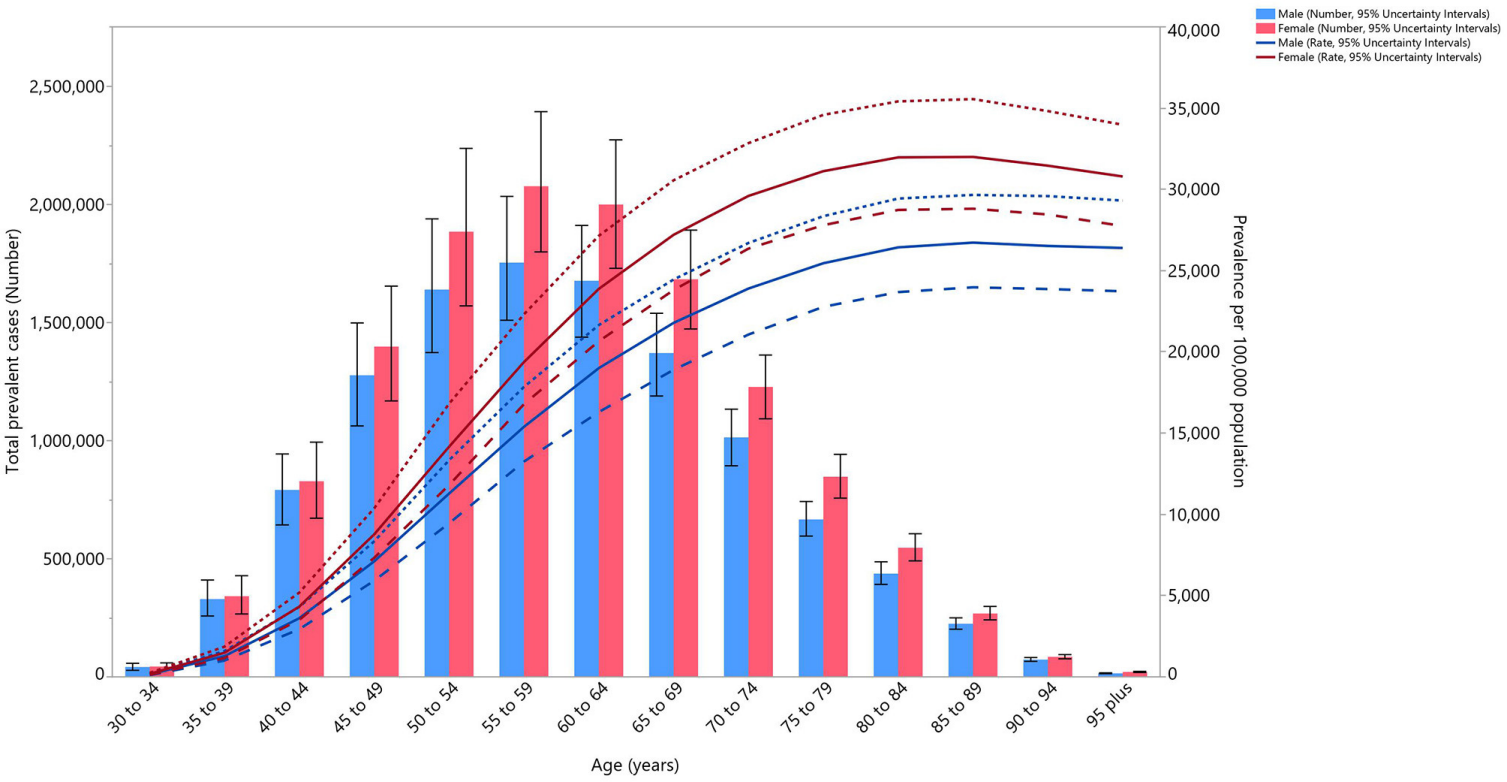
Numbers of prevalent cases and prevalence of osteoarthritis per 100,000 population in the Middle East and North Africa region, by age and sex in 2019. Dotted and dashed lines indicate 95% upper and lower uncertainty intervals, respectively (generated from data available from http://ghdx.healthdata.org/gbd-results-tool).

In 2019, the total number of prevalent cases of OA was higher in females. The number of cases also increased with advancing age, for both sexes, peaking in those aged between 55 and 59 years, before decreasing again. The estimated prevalence per 100,000 population peaked in the 80–84 age group, but then reached a plateau in both sexes. However, the higher prevalence among women in the different age groups was not substantially higher than among men (Figure 1).

The total incident cases of OA in 2019 was higher among women than in men, but the difference was not substantial. The number of incident cases was highest in the 45–49 age group, and then decreased with age. The incidence rate of OA peaked in the 55–59 age group, for both sexes, and then decreased with increasing age (Supplementary Figure S7).

The total YLDs followed a similar pattern to the incidence and prevalence, that being higher YLDs for females than males, but the difference again was not substantial. Total YLDs reached their peak in the 55–59 age group, and then decreased with increased age. YLD rates also increased with age until they reached a peak in the 75–79 age group, and then slowly decreased with increasing age (Supplementary Figure S8).

**Figure 2:**
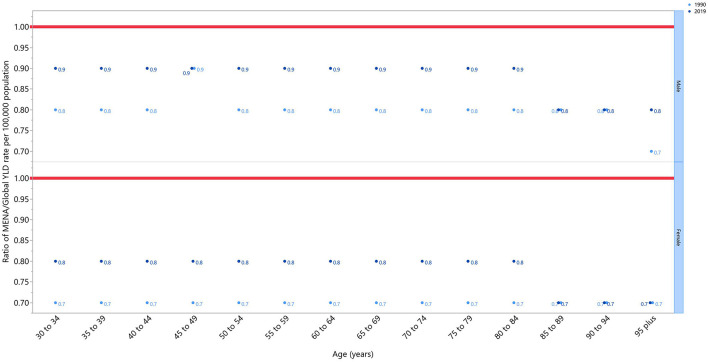
Ratio of the Middle East and North Africa region to the global osteoarthritis YLD rate according to age group and sex, 1990–2019. YLD, years lived with disability (generated from data available from http://ghdx.healthdata.org/gbd-results-tool).

The rate ratio, comparing the age-standardized YLD rates in MENA to the global rates for each age groups and sex, in 1990 and 2019, showed a generally increasing pattern. This ratio increased by 0.1 per 100,000 population, in both sexes and in almost all age groups, from 1990 to 2019, reaching almost 0.9 in men, and 0.8 in women. The only exceptions in this pattern were for age groups more than 85, which remained at 0.7 for females and 0.8 for males, and the 45–49 age group in men, which was 0.9 in both 1990 and 2019. It is important to note that the MENA to global ratio was lower than 1 in all the age groups, meaning that the overall burden of OA in MENA was lower than the global average (Figure 2).

### Affected Joints

**Figure 3:**
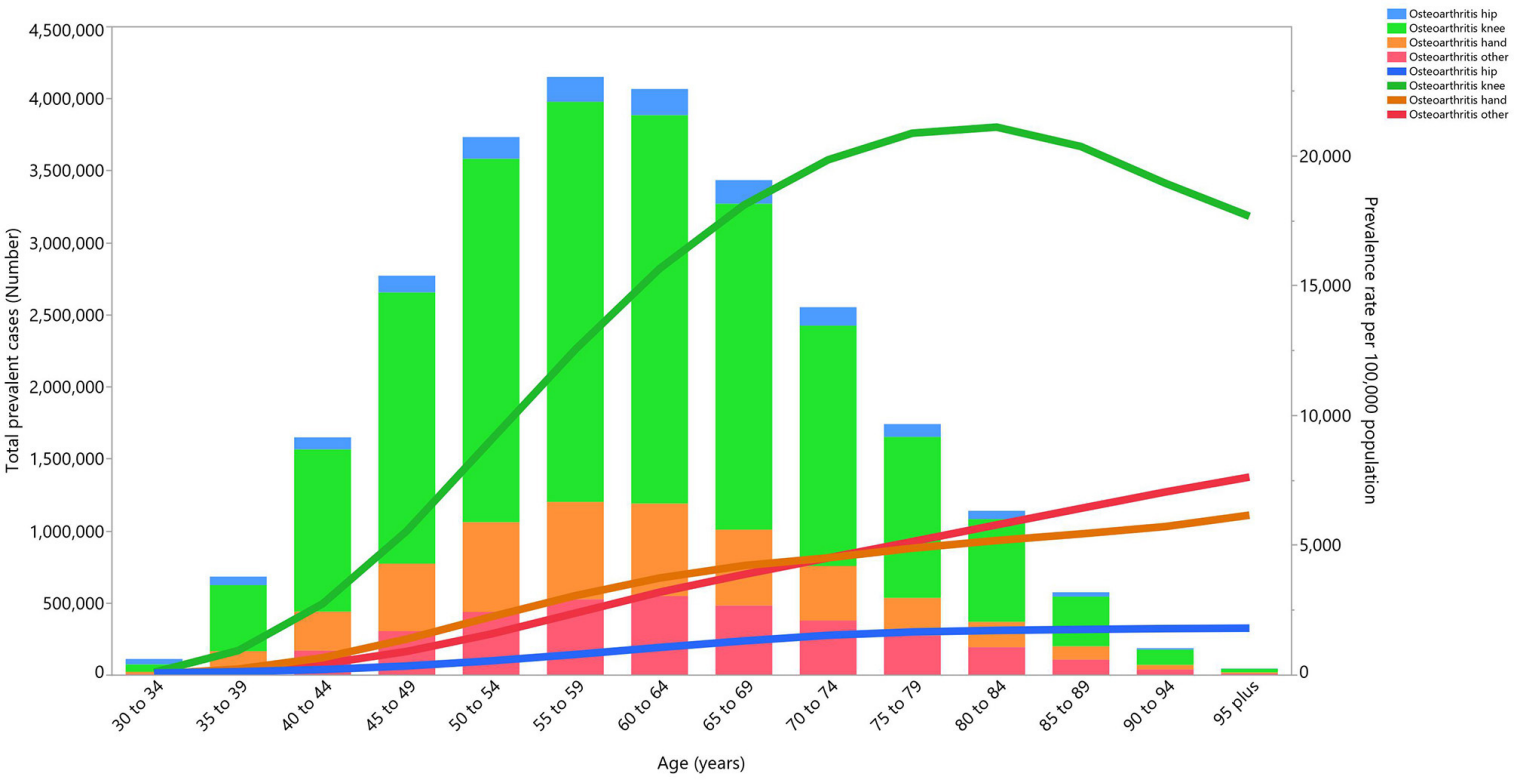
Numbers of prevalent cases and prevalence for osteoarthritis per 100,000 population in the Middle East and North Africa region, by age and cause in 2019 (generated from data available from http://ghdx.healthdata.org/gbd-results-tool).

Almost all of the joints affected by OA followed a similar pattern to the total prevalence, as their number of prevalent cases increased with age until reaching a peak in the 55–59 age group, and then decreasing with increasing age. Knee OA accounted for the most cases of OA, followed by hand OA and hip OA. The prevalence of these types of OA also increased with age. Knee OA reached its peak in the 80–84 age group, while the other types of OA continued to increase in a linear fashion (Figure 3).

The total incident cases of OA increased with age, until reaching a peak in those aged between 55 and 59 years, and then decreased with age. The number of incident cases of hand OA followed this decline up to the 70–74 age group, before increasing slowly until the final age group. The most commonly involved joints, through all age groups, were the knee and hand. The incidence rates of all types of OA peaked in the 45–49 age group, and then decreased substantially (Supplementary Figure S9).

The YLD patterns for the different joints followed an identical trend to the prevalence. The peak YLD for all forms of OA was in the 55–59 age group, and then there was a substantial decline for the remaining age groups. The largest proportion of the YLDs were caused by OA of the knee and hand. The YLD rates for all OA types increased in a linear manner with age, except for knee OA, which peaked in the 75–79 age group (Supplementary Figure S10).

### Association Between the OA Burden and the Socio-demographic Index (SDI)

**Figure 4:**
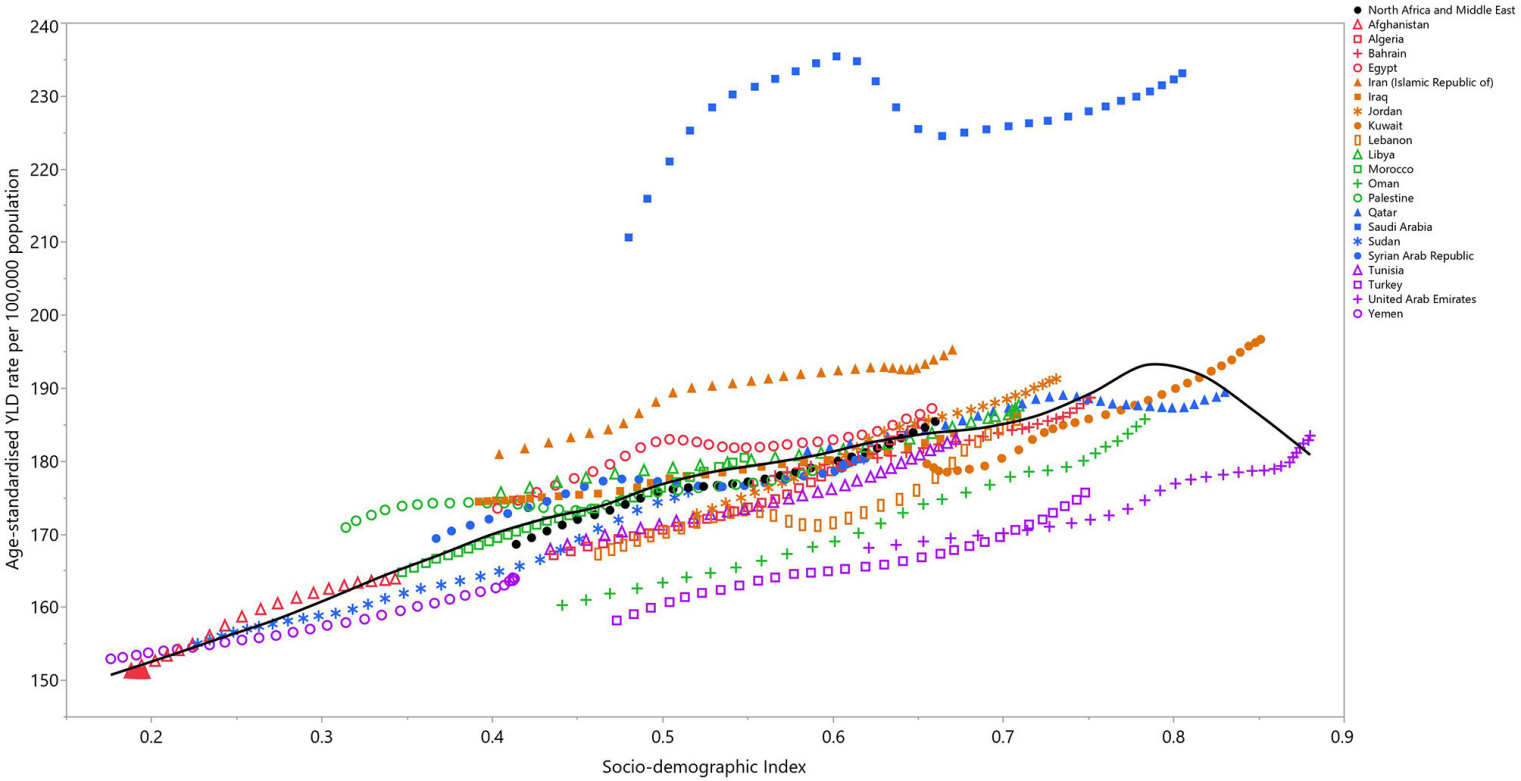
Age-standardized YLD rates of osteoarthritis for 21 countries and territories, by SDI in 2019; Expected values based on the Socio-demographic Index and disease rates in all locations are shown as the black line. Each point shows the observed age-standardized YLD rate for each country in 2019. YLD, years lived with disability; SDI, Socio-demographic Index (generated from data available from http://ghdx.healthdata.org/gbd-results-tool).

In 2019, a positive relationship was observed between the SDI and the age-standardized YLD per 100,000 population at the national level. After a linear increase up to an SDI of 0.8, the mean ASR of the YLDs started to decrease. Saudi Arabia, Iran, Jordan, and Egypt had a higher than expected burden, while Turkey, the United Arab Emirates, and Oman had a lower than expected burden (Figure 4).

## Discussion

The present study provides the most recently estimated prevalence, incidence, and YLDs, alongside their representative age-standardized rates, for the 21 countries in the MENA region from 1990 to 2019. In 2019, the burden of OA in this region consisted of 24.6 million prevalent cases, 2.3 million incident cases, and more than 852,000 YLDs. Generally, countries with the highest prevalence (i.e., Saudi Arabia, Kuwait, and Iran), also had the highest incidence and YLD rates. Similarly, countries with the lowest prevalence (i.e., Yemen and Afghanistan) also had the lowest incidence and YLD rates.

This article delivers an in-depth national-level estimation of the OA burden by sex and age. This research also provides a proportional estimation for the individual joints affected, by age and sex in the countries that comprise the MENA region. It is important to note that the prevalence, incidence, and YLDs of OA have not previously been reported at the national-level in the MENA region, with the most recent study in this area being conducted to estimate the burden of musculoskeletal disorders using data from the 2013 GBD Study (16).

Compared to the previously published global burden of OA, a report from the GBD 2017 study (10), the age-standardized prevalence of OA increased from 4,609.8 in 2017 to 5,342.8 in 2019. In contrast, the ASR percentage decreased from 12.8 to 9.3. The age-standardized incidence also increased substantially, from 231 to 430.4, from 2017 to 2019, but their respective percentage changes decreased from 12 to 9.4%. Finally, the ASR of the YLDs increased from 144.8 to 185.4 and the percentage change dropped from 13.4 to 10% over the measurement period (10). The observed increasing prevalence, incidence, and YLDs, from 1990 to 2019, were similar to previously reported studies (10, 16). The ASR of the prevalence, incidence and YLDs are expected to continue to rise in the future, increasing the burden of OA globally and highlighting the importance of preventive measures to halt this increasing burden (10).

Although this study measures and reports the trends of OA from 1990 to 2019, the interpretation of country level data, especially in a specific year, should be done cautiously. As shown in Supplementary Table S1, Yemen, Afghanistan, and Sudan have the lowest prevalence in the region of MENA; but the data corresponding to this condition may be highly biased due to possible healthcare impairments imposed by war and governmental imbalance (17). These situations may direct most of the healthcare resources to managing more acute situations rather than chronic diseases such as OA (17). Furthermore, as most of these are considered low income countries, people with chronic conditions may not seek medical treatment for their condition until a health emergency occurs. Furthermore, the demographic data from these countries are prone to bias, as a considerable proportion of the population may have migrated to other countries, as refugees of war, and those remaining are rarely the subjects of any form of demographic study (18). A similar argument can be made for the surprisingly low age-standardized prevalence of OA in Iraq and Palestine, which might be due to lower detection rates, or a higher mortality in these countries in the condition of war. Sociodemographic differences may be another explanation for the lower prevalence of OA in the aforementioned countries. According to the 2020 population yearbook of the United Nations, urbanization, which is an indicative factor in healthcare access, was significantly lower in these countries, with rates of 23.5% in Afghanistan, 28.9% in Yemen, and 35.3% in Sudan (18). The estimated life expectancy at birth in these three countries was also lower than in other similar MENA countries. For example, in Afghanistan the life expectancy at birth for men was 45 and for women it was 44 years old. Furthermore, in these countries the majority of the population is under 40 years old, with the peak age group being 5–9 years old (except in Yemen, where it was the 0–4 age group) (18).

In 2019, the prevalence estimate of OA (per 100,000) in MENA peaked in the 80–84 age group for both sexes, but this peak was in a lower age group than that found in the previous GBD report (10). The peak in this age group may be due in part to a lower level of bone mineral density, frequent fractures, and the loss of muscular support at the joints in this age group. Furthermore, the lowered age in the peak prevalence may be explained by a more sedentary lifestyle, low levels of vitamin D, an increased prevalence of osteoporosis and of fractures, and the trend toward higher body mass index (BMI), leading to an earlier onset and OA peaking in a younger age group (19–21).

In agreement with the previous global report, in 2019 the incidence rate of OA in MENA peaked in the 55–59 age group, and decreased in a linear manner with increasing age (10). The positive relationship found between SDI and OA in previous research was also observed in the present study (10). Countries, such as Saudi Arabia and Iran, had much higher age-standardized YLDs than other MENA countries at the same SDI level. The aforementioned association was also seen in the middle to low SDI countries, such as Yemen, Sudan, Afghanistan, and Palestine. The YLDs increases were much larger at lower SDI levels than they were for higher SDI levels (22). In countries with high SDIs, a more sedentary lifestyle, a higher BMI score, and a longer life span would be expected, which can all lead to a higher OA burden. The drop in the age-standardized YLDs, as found here after an SDI score of 0.8, has also been seen worldwide (22). This trend may be indicative of better healthcare access or better healthcare quality in countries with SDIs higher than 0.8.

Previous research has found (10) the burden of OA to be higher among women than among men, which is in accordance with our findings. This higher burden can be explained by several underlying causes, including post-menopausal hormonal changes that lead to early osteopenia and osteoporosis, which also affects the biomechanical structure of the joints. Another explanation for this difference might be the generally stronger joint support in men, as they mostly have more muscle bulk and stronger ligaments. These differences can lead to more traumatic fractures and the imposition of higher joint stress in women, when compared to men (20). Women also tend to have higher BMI than men, which is one of the most important modifiable differences between the two genders. With this in mind, preventive strategies to reduce weight should be more personalized and more clearly targeted at those with high BMI (10, 23).

This study is the first to report the proportion of OA according to the joint(s) affected and by age group. This investigation showed that knee OA, with an incidence peak in the 45–49 age group, was the most common type of OA in the MENA region. Furthermore, most of the YLDs (per 100,000 population) were due to knee OA, which peaked in the 75–79 age group, and was followed by hand OA, which peaked in the 95 plus age group. Alongside the devastating burden of knee OA, the present research also highlights the need to take a more preventative approach, especially for hand OA, which does not have the end of care option of arthroplastic surgery.

Pain and disability are the most common complaints among patients with OA, and so pain management is considered to be one of the most important targets of medical therapy (24). There are multiple pain-relieving treatments suggested for OA, with the first line being non-pharmacological therapies, such as specific exercises, splinting, and thermal therapies, but the results of these treatments are mostly mixed and inconclusive (25). Although there are findings that suggest pharmacological therapy is the most promising approach to pain relief, the long-term effects and the effects of these treatments remain controversial (26). The progressive disability and aggravating pain persuades most patients to undergo joint replacement therapy (27). Arthroplasty is the end of care treatment for OA, an expensive method which can only be undertaken effectively on a limited group of joints, such as the hip and knee, but this condition still lacks a definitive cure (4). Moreover, it should be noted that in order to be successful, surgical treatments not only require an experienced surgeon, but also a suitable surgical strategy and high-quality materials. Without the previously mentioned conditions, surgical complications and more pain are inevitable (28, 29).

OA imposes very large costs on both the patients and the healthcare system, with the costs being both direct (e.g., medical and surgical treatments) and indirect (e.g., mental status management and premature retirement). These costs are mainly out of the patient's pocket and can be catastrophic in a low-income household (8). OA is also considered to be a substantial cause of unmet medical needs (30). This highlights the need for better preventive measures, rather than a breakthrough treatment (1).

Modifiable risk factors are the most crucial targets of OA prevention and include high BMI, physical inactivity, trauma, joint mal-positioning, and chronic mechanical stress. Previous research has shown that the most supported preventative methods are BMI reductions, increased physical activity, the prevention of trauma, and avoiding continuous chronic joint stress (31, 32). Several reports have shown that physical activity is much lower in MENA countries, than the global average, and is lower in women than in men, although physical activity has started to rise in recent decades (33, 34). Obesity on the other hand, has increased in recent years, and with the recent shift in diets toward more processed food, obesity is expected to continue to increase (33). The first and most effective interventions in the early stages of OA are lifestyle alterations, such as increasing physical activity and weight reduction. As well as being effective preventive strategies, these lifestyle interventions should also be made for almost all OA patients (1).

BMI, which is one of the most important modifiable risk factors of OA, is increasing globally and most dramatically in developing countries, including in the MENA region (21, 33). This highlights the need for the development of preventative measures which focus on reducing BMI, in order to limit the burden of OA. Countries such as Kuwait, which had a high average BMI in the region and a high YLD attributed to BMI, also had a high OA burden, confirming this necessity. Non-pharmacological therapies remain the most cost-effective measure for prevention, management, and even treating OA, especially in the knee and hip joints (35). Several studies suggest that lowering BMI scores, and increased physical activity are associated with significant reductions in pain and an increased quality of life (1, 20, 35). It should also be noted that although OA may seem to be inevitable with increased age, it can be halted and/or postponed with the adoption of the previously mentioned lifestyle changes. These interventions are most effective if they are implemented in the early years of childhood, as the changes in diet and an increased sedentary lifestyle appear to suggest there will be further increases in the mean BMI over the coming years (33).

Since GBD studies encompass a large number of mostly heterogenic studies with different diagnostic methods (and sometimes these are different for each joint), a simpler and more generalizable diagnostic method must be used. The Kellgren-Lawrence criteria, which was initially a severity classification system, is applicable in almost every OA study, as it utilizes x-ray evidence (36, 37). The Kellgren-Lawrence system promises a rather simple and objective definition of OA severity in a wide range of joints, making it an appropriate classification method for epidemiological studies, such as the GBD. Although using this classification system strongly increases the specificity of this study, it reduces its sensitivity by confining the cases to grades 2–4. By the aforementioned limitation, the initial cases, and milder forms of OA (i.e., grades 0 and 1) are not included in this study, indicating that the current results can merely be an underestimation of the true burden of OA in the region of MENA.

## Conclusions

The prevalence of OA is increasing globally and the MENA region is no exception to this pattern. This disease is generally more prevalent among women, has a positive association with age and SDI, and most commonly affects the knee, hip and hand joints. As populations are aging and the risk factors of OA, such as high BMI, are becoming more widespread, the burden of OA is expected to continue to increase. Preemptive measures should be developed to target the risk factors and to allow the early diagnosis of this condition. Lifestyle changes and increasing knowledge regarding OA are among the most effective preventative policies. Increasing physical activity (both through exercise and reducing a sedentary lifestyle), following a healthy diet, and controlling BMI, are proven preventive and treatment strategies that should be implemented at a young age. However, these interventions do not obviate the need for better healthcare systems and patient management strategies. A regular analysis of the prevalence and incidence of OA is required to provide further assessments of the trends associated with this disease and to monitor the success rate of prevention policies.

## Data Availability Statement

Publicly available datasets were analyzed in this study. This data can be found here: http://ghdx.healthdata.org/gbd-results-tool.

## Ethics Statement

The present report was reviewed and approved by the Ethics Committee of Tabriz University of Medical Sciences, Tabriz, Iran (IR.TBZMED.REC.1400.1167).

## Author Contributions

SS, A-AK, and MA designed the study. AS, SN, and SS analyzed the data and performed the statistical analyses. AS, SN, MS, JK, and GC drafted the initial manuscript. All authors reviewed the drafted manuscript for critical and approved the final version of the manuscript.

## Funding

The Bill and Melinda Gates Foundation, who were not involved in any way in the preparation of this manuscript, funded the GBD study. The Tabriz University of Medical Sciences, Tabriz, Iran (Grant No. 68352) also supported the present report.

## Author Disclaimer

This study is based on publicly available data and solely reflects the opinion of its authors and not that of the Institute for Health Metrics and Evaluation.

## Conflict of Interest

The authors declare that the research was conducted in the absence of any commercial or financial relationships that could be construed as a potential conflict of interest.

## Publisher's Note

All claims expressed in this article are solely those of the authors and do not necessarily represent those of their affiliated organizations, or those of the publisher, the editors and the reviewers. Any product that may be evaluated in this article, or claim that may be made by its manufacturer, is not guaranteed or endorsed by the publisher.

## Supplementary Material

Supplementary Figure S1 Age-standardized point prevalence of osteoarthritis (per 100,000 population) in the Middle East and North Africa region in 2019, by sex and country (generated from data available from http://ghdx.healthdata.org/gbd-results-tool).

Supplementary Figure S2 The percentage change in the age-standardized point prevalence of osteoarthritis in the Middle East and North Africa region from 1990 to 2019, by sex and country (generated from data available from http://ghdx.healthdata.org/gbd-results-tool).

Supplementary Figure S3 Age-standardized incidence rate of osteoarthritis (per 100,000 population) in the Middle East and North Africa region in 2019, by sex and country (generated from data available from http://ghdx.healthdata.org/gbd-results-tool).

Supplementary Figure S4 The percentage change in the age-standardized incidence of osteoarthritis in the Middle East and North Africa region from 1990 to 2019, by sex and country (generated from data available from http://ghdx.healthdata.org/gbd-results-tool).

Supplementary Figure S5 Age-standardized YLDs rate of osteoarthritis (per 100,000 population) in the Middle East and North Africa region in 2019, by sex and country. YLD, years lived with disability (generated from data available from http://ghdx.healthdata.org/gbd-results-tool).

Supplementary Figure S6 The percentage change in the age-standardized YLDs of osteoarthritis in the Middle East and North Africa region from 1990 to 2019, by sex and country. YLD, years lived with disability (generated from data available from http://ghdx.healthdata.org/gbd-results-tool).

Supplementary Figure S7 Numbers of incident cases and incidence rate of osteoarthritis per 100,000 population in the Middle East and North Africa region, by age and sex in 2019; Dotted and dashed lines indicate 95% upper and lower uncertainty intervals, respectively (generated from data available from http://ghdx.healthdata.org/gbd-results-tool).

Supplementary Figure S8 Numbers of YLDs and YLD rate of osteoarthritis per 100,000 population in the Middle East and North Africa region, by age and sex in 2019; Dotted and dashed lines indicate 95% upper and lower uncertainty intervals, respectively. YLD, years lived with disability (generated from data available from http://ghdx.healthdata.org/gbd-results-tool).

Supplementary Figure S9 Number of incident cases and incidence rate for osteoarthritis per 100,000 population in the Middle East and North Africa region, by age and cause in 2019 (generated from data available from http://ghdx.healthdata.org/gbd-results-tool).

Supplementary Figure S10 Number of YLDs and YLD rate for osteoarthritis per 100,000 population in the Middle East and North Africa region, by age and cause in 2019. YLD= years lived with disability (generated from data available from http://ghdx.healthdata.org/gbd-results-tool).

Supplementary Table S1 Prevalence of osteoarthritis in 1990 and 2019 for both sexes and percentage change in age-standardized rates (ASRs) per 100,000 in the North Africa and the Middle East region (generated from data available from http://ghdx.healthdata.org/gbd-results-tool).

Supplementary Table S2 Incidence of osteoarthritis in 1990 and 2019 for both sexes and percentage change in age-standardized rates (ASRs) per 100,000 in the North Africa and the Middle East region (generated from data available from http://ghdx.healthdata.org/gbd-results-tool).

Supplementary Table S3 YLDs due to osteoarthritis in 1990 and 2019 for both sexes and percentage change in age-standardized rates (ASRs) per 100,000 in the North Africa and the Middle East region (generated from data available from http://ghdx.healthdata.org/gbd-results-tool).

## References

[1] Hunter DJ Bierma-Zeinstra S. Osteoarthritis. Lancet. (2019) 393:1745–59. 10.1016/S0140-6736(19)30417-9 31034380

[2] Bacon K LaValley MP Jafarzadeh SR Felson D. Does cartilage loss cause pain in osteoarthritis and if so, how much? Ann Rheum Dis. (2020) 79:1105–10. 10.1136/annrheumdis-2020-217363 32381567 PMC10406023

[3] Pereira D Peleteiro B Araújo J Branco J Santos RA Ramos E. The effect of osteoarthritis definition on prevalence and incidence estimates: a systematic review. Osteoarthritis Cartilage. (2011) 19:1270–85. 10.1016/j.joca.2011.08.009 21907813

[4] Katz JN Arant KR Loeser RF. Diagnosis and treatment of hip and knee osteoarthritis: a review. JAMA. (2021) 325:568–78. 10.1001/jama.2020.22171 33560326 PMC8225295

[5] Glyn-Jones S Palmer AJ Agricola R Price AJ Vincent TL Weinans H. Osteoarthritis. Lancet. (2015) 386:376–87. 10.1016/S0140-6736(14)60802-3 25748615

[6] Sacitharan PK. Ageing and osteoarthritis. Subcell Biochem. (2019) 91:123–59. 10.1007/978-981-13-3681-2_6 30888652

[7] Mobasheri A Fonseca JE Gualillo O Henrotin Y Largo R Herrero-Beaumont G. Editorial: inflammation and biomarkers in osteoarthritis. Front Med. (2021) 8:727700. 10.3389/fmed.2021.727700 34386512 PMC8353120

[8] Zhao X Shah D Gandhi K Wei W Dwibedi N Webster L. Clinical, humanistic, and economic burden of osteoarthritis among noninstitutionalized adults in the United States. Osteoarthr Cartil. (2019) 27:1618–26. 10.1016/j.joca.2019.07.002 31299387

[9] Al-Saleh JA Albelooshi AA Salti AA Farghaly M Ghorab AM Linga S. Burden, treatment patterns and unmet needs of osteoarthritis in Dubai: a retrospective analysis of the Dubai Real-World Claims Database. Rheumatol Therapy. (2021) 9:151—74. 10.1007/s40744-021-00391-z 34784014 PMC8814126

[10] Safiri S Kolahi A-A Smith E Hill C Bettampadi D Mansournia MA. Global, regional and national burden of osteoarthritis 1990-2017: a systematic analysis of the Global Burden of Disease Study 2017. Ann Rheum Dis. (2020) 79:819–28. 10.1136/annrheumdis-2019-216515 32398285

[11] Driban JB Harkey MS Barbe MF Ward RJ MacKay JW Davis JE. Risk factors and the natural history of accelerated knee osteoarthritis: a narrative review. BMC Musculoskelet Disord. (2020) 21:1–11. 10.1186/s12891-020-03367-2 32471412 PMC7260785

[12] Vina ER Kwoh CK. Epidemiology of osteoarthritis: literature update. Curr Opin Rheumatol. (2018) 30:160–7. 10.1097/BOR.0000000000000479 29227353 PMC5832048

[13] Prieto-Alhambra D Judge A Javaid MK Cooper C Diez-Perez A Arden NK. Incidence and risk factors for clinically diagnosed knee, hip and hand osteoarthritis: influences of age, gender and osteoarthritis affecting other joints. Ann Rheum Dis. (2014) 73:1659–64. 10.1136/annrheumdis-2013-203355 23744977 PMC3875433

[14] Plotz B Bomfim F Sohail MA Samuels J. Current epidemiology and risk factors for the development of hand osteoarthritis. Curr Rheumatol Rep. (2021) 23:61. 10.1007/s11926-021-01025-7 34216294

[15] Vos T Lim SS Abbafati C Abbas KM Abbasi M Abbasifard M. Global burden of 369 diseases and injuries in 204 countries and territories, 1990-2019: a systematic analysis for the Global Burden of Disease Study 2019. Lancet. (2020) 396:1204–22. 10.1016/S0140-6736(20)30925-9 33069326 PMC7567026

[16] Moradi-Lakeh M Forouzanfar MH Vollset SE El Bcheraoui C Daoud F Afshin A. Burden of musculoskeletal disorders in the Eastern Mediterranean Region, 1990–2013: findings from the Global Burden of Disease Study 2013. Ann Rheum Dis. (2017) 76:1365–73. 10.2337/dc16-1075 28209629 PMC5738600

[17] Raad II Chaftari A-M Dib RW Graviss EA Hachem R. Emerging outbreaks associated with conflict and failing healthcare systems in the Middle East. Infect Control Hosp Epidemiol. (2018) 39:1230–6. 10.1017/ice.2018.177 30099975

[18] United Nations. Demographic Yearbook 2020. 71 ed. Geneva: The TDoEaSAo, Secretariat UN, United Nations Publication (2021).

[19] Zafeiris EP Babis GC Zafeiris CP Chronopoulos E. Association of vitamin D, BMD and knee osteoarthritis in postmenopausal women. J Musculoskelet Neuronal Interact. (2021) 21:509–16. 34854390 PMC8672405

[20] Geusens PP van den Bergh JP. Osteoporosis and osteoarthritis: shared mechanisms and epidemiology. Curr Opin Rheumatol. (2016) 28:97–103. 10.1097/BOR.0000000000000256 26780427

[21] Lin X Xu Y Xu J Pan X Song X Shan L. Global burden of noncommunicable disease attributable to high body mass index in 195 countries and territories, 1990–2017. Endocrine. (2020) 69:310–20. 10.1007/s12020-020-02352-y 32488838

[22] Hunter DJ March L Chew M. Osteoarthritis in 2020 and beyond: a Lancet Commission. Lancet. (2020) 396:1711–2. 10.1016/S0140-6736(20)32230-3 33159851

[23] Misra D Fielding RA Felson DT Niu J Brown C Nevitt M. Risk of knee osteoarthritis with obesity, sarcopenic obesity, and sarcopenia. Arthritis Rheumatol. (2019) 71:232–7. 10.1002/art.40692 30106249 PMC6374038

[24] Vaughn IA Terry EL Bartley EJ Schaefer N Fillingim RB. Racial-Ethnic differences in osteoarthritis pain and disability: a meta-analysis. J Pain. (2019) 20:629–44. 10.1016/j.jpain.2018.11.012 30543951 PMC6557704

[25] Kroon FPB Carmona L Schoones JW Kloppenburg M. Efficacy and safety of non-pharmacological, pharmacological and surgical treatment for hand osteoarthritis: a systematic literature review informing the 2018 update of the EULAR recommendations for the management of hand osteoarthritis. RMD Open. (2018) 4:e000734. 10.1136/rmdopen-2018-000734 30402266 PMC6203105

[26] Gregori D Giacovelli G Minto C Barbetta B Gualtieri F Azzolina D. Association of pharmacological treatments with long-term pain control in patients with knee osteoarthritis: a systematic review and meta-analysis. JAMA. (2018) 320:2564–79. 10.1001/jama.2018.19319 30575881 PMC6583519

[27] Neuprez A Neuprez AH Kaux J-F Kurth W Daniel C Thirion T. Total joint replacement improves pain, functional quality of life, and health utilities in patients with late-stage knee and hip osteoarthritis for up to 5 years. Clin Rheumatol. (2020) 39:861–71. 10.1007/s10067-019-04811-y 31720892

[28] Pincus D Jenkinson R Paterson M Leroux T Ravi B. Association between surgical approach and major surgical complications in patients undergoing total hip arthroplasty. JAMA. (2020) 323:1070–6. 10.1001/jama.2020.0785 32181847 PMC7078797

[29] Wilson HA Middleton R Abram SGF Smith S Alvand A Jackson WF. Patient relevant outcomes of unicompartmental versus total knee replacement: systematic review and meta-analysis. BMJ. (2019) 364:l352. 10.1136/bmj.l352 30792179 PMC6383371

[30] Jo H Kim E-s Jung B Sung S-H Ha I-H. Association between osteoarthritis and unmet medical needs in Korea: limitations in activities as a mediator. BMC Public Health. (2020) 20:1026. 10.1186/s12889-020-09140-3 32600311 PMC7325304

[31] Whittaker JL Runhaar J Bierma-Zeinstra S Roos EM. A lifespan approach to osteoarthritis prevention. Osteoarthr. Cartil. (2021) 29:1638–653. 10.1016/j.joca.2021.06.015 34560260

[32] He Y Li Z Alexander PG Ocasio-Nieves BD Yocum L Lin H. Pathogenesis of osteoarthritis: risk factors, regulatory pathways in chondrocytes, and experimental models. Biology. (2020) 9:194. 10.3390/biology9080194 32751156 PMC7464998

[33] Azizi F Hadaegh F Hosseinpanah F Mirmiran P Amouzegar A Abdi H. Metabolic health in the Middle East and North Africa. Lancet Diab Endocrinol. (2019) 7:866–79. 10.1016/S2213-8587(19)30179-2 31422063

[34] Chaabane S Chaabna K Abraham A Mamtani R Cheema S. Physical activity and sedentary behaviour in the Middle East and North Africa: an overview of systematic reviews and meta-analysis. Sci Rep. (2020) 10:9363. 10.1038/s41598-020-66163-x 32518254 PMC7283267

[35] Nelson AE. Osteoarthritis year in review 2017: clinical. Osteoarthritis Cartilage. (2018) 26:319–25. 10.1016/j.joca.2017.11.014 29229563 PMC5835411

[36] Kellgren JH Lawrence JS. Radiological assessment of osteo-arthrosis. Ann Rheum Dis. (1957) 16:494–502. 10.1136/ard.16.4.494 13498604 PMC1006995

[37] Kellgren JH. Radiological signs of rheumatoid arthritis; a study of observer differences in the reading of hand films. Ann Rheum Dis. (1956) 15:55–60. 10.1136/ard.15.1.55 13303060 PMC1006857

