# Supplementary figures and images for: The Burden of Osteoarthritis in the Middle East and North Africa Region From 1990 to 2019
Source: Front Med (Lausanne). 2022 Jun 23;9:881391. doi: 10.3389/fmed.2022.881391 (PMC9261477; doi:10.3389/fmed.2022.881391)

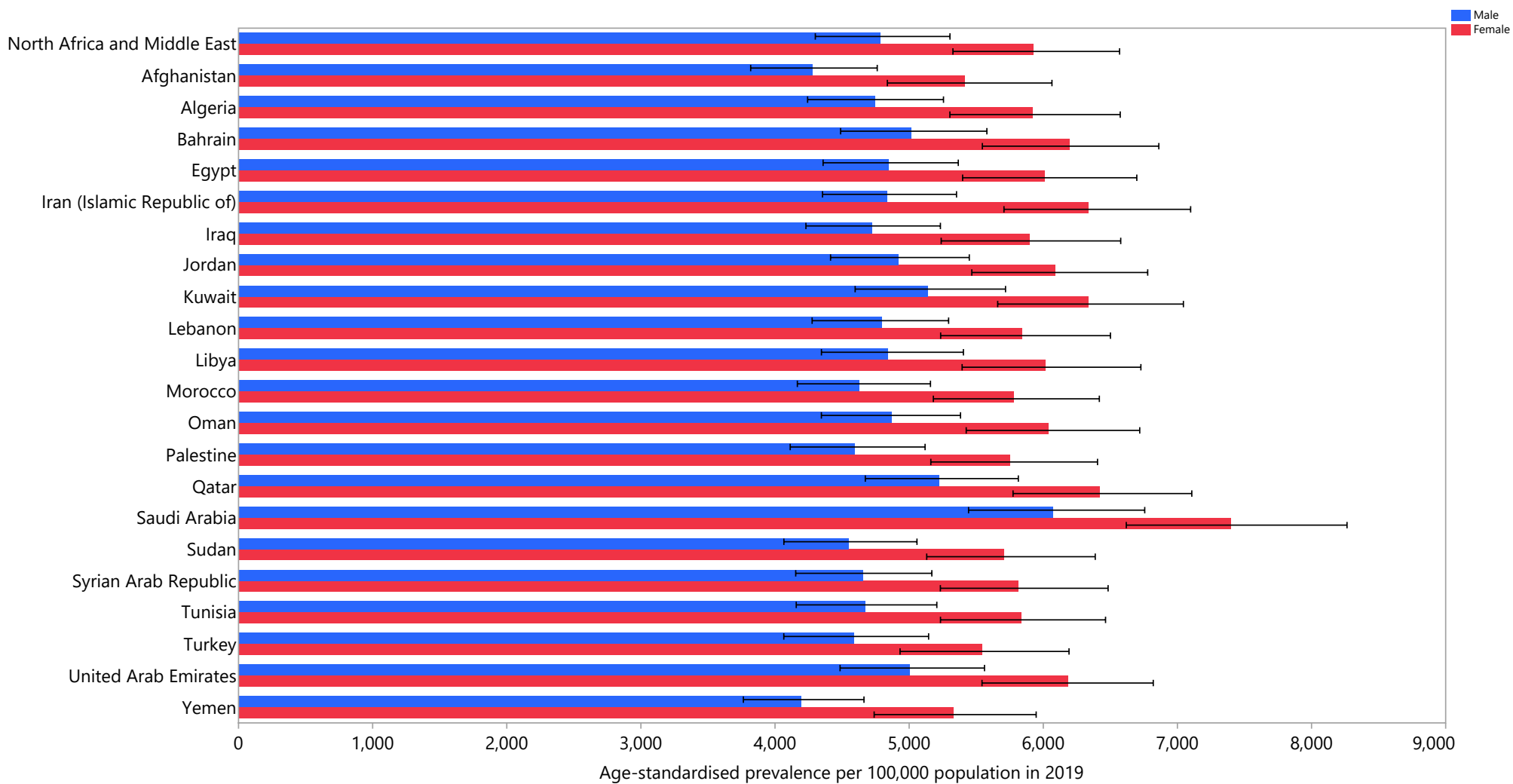

Supplement: Supplementary Figure S1 — Age-standardized point prevalence of osteoarthritis (per 100,000 population) in the Middle East and North Africa region in 2019, by sex and country (generated from data available from http://ghdx.healthdata.org/gbd-results-tool). [file Image_1.PDF]

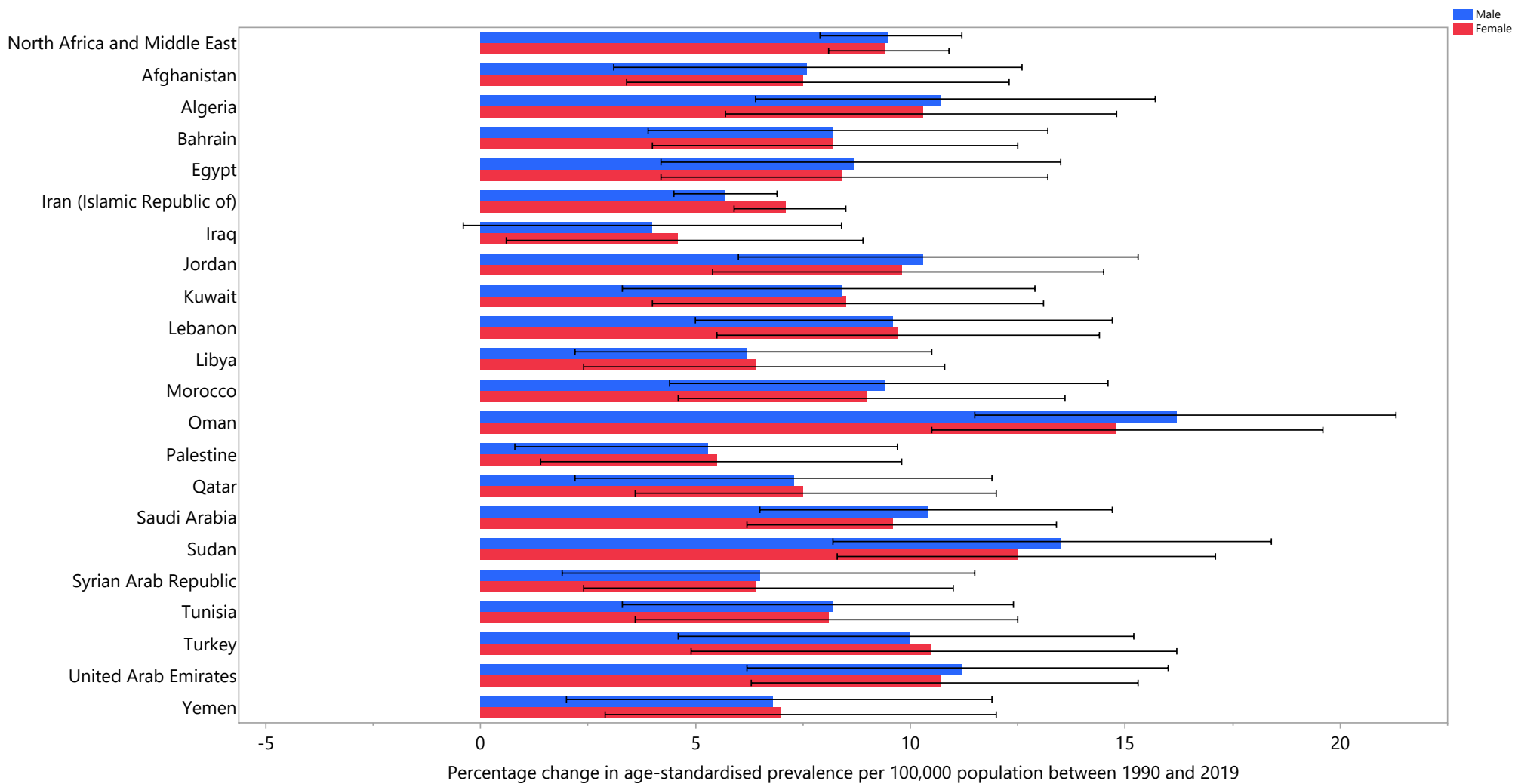

Supplement: Supplementary Figure S2 — The percentage change in the age-standardized point prevalence of osteoarthritis in the Middle East and North Africa region from 1990 to 2019, by sex and country (generated from data available from http://ghdx.healthdata.org/gbd-results-tool). [file Image_2.PDF]

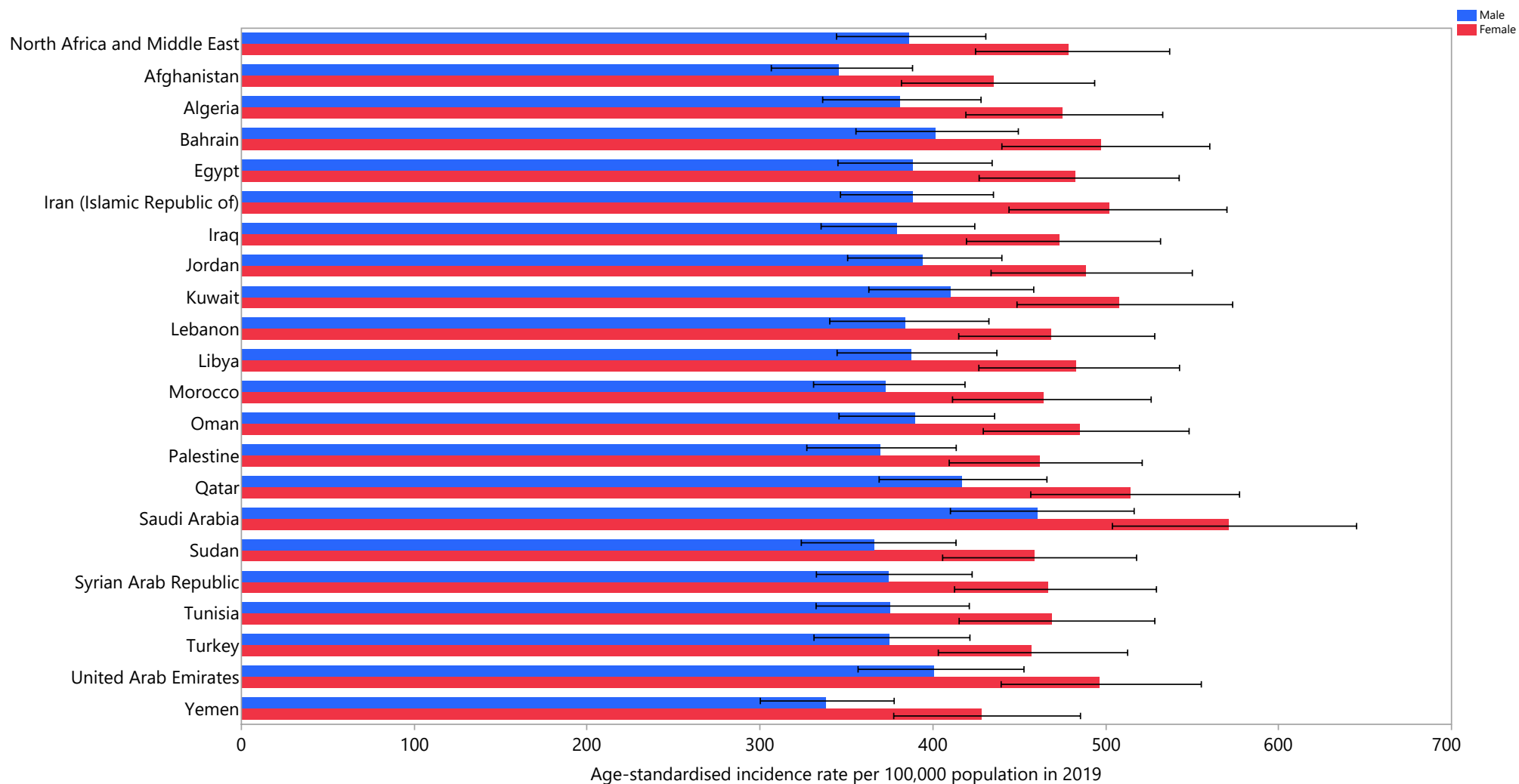

Supplement: Supplementary Figure S3 — Age-standardized incidence rate of osteoarthritis (per 100,000 population) in the Middle East and North Africa region in 2019, by sex and country (generated from data available from http://ghdx.healthdata.org/gbd-results-tool). [file Image_3.PDF]

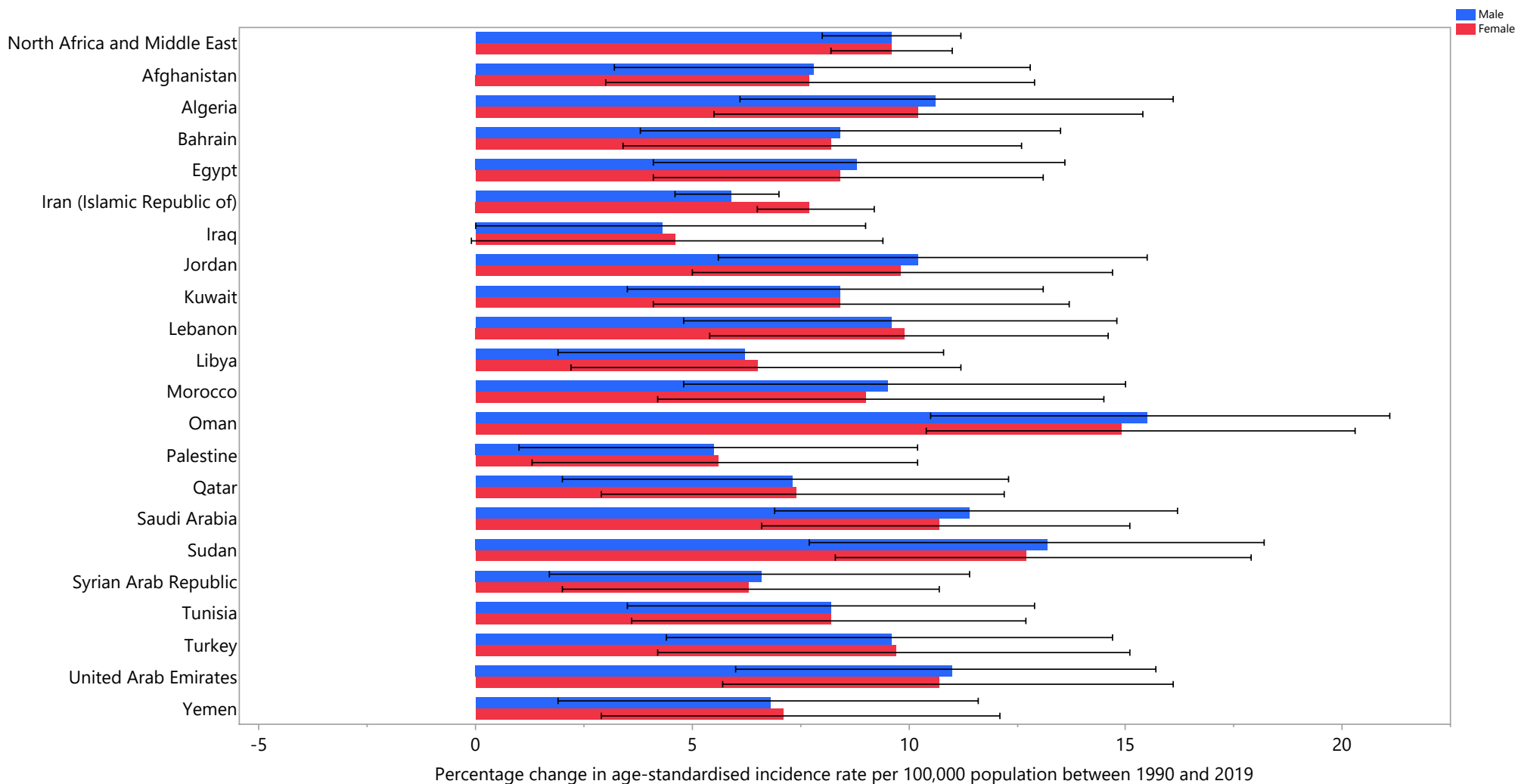

Supplement: Supplementary Figure S4 — The percentage change in the age-standardized incidence of osteoarthritis in the Middle East and North Africa region from 1990 to 2019, by sex and country (generated from data available from http://ghdx.healthdata.org/gbd-results-tool). [file Image_4.PDF]

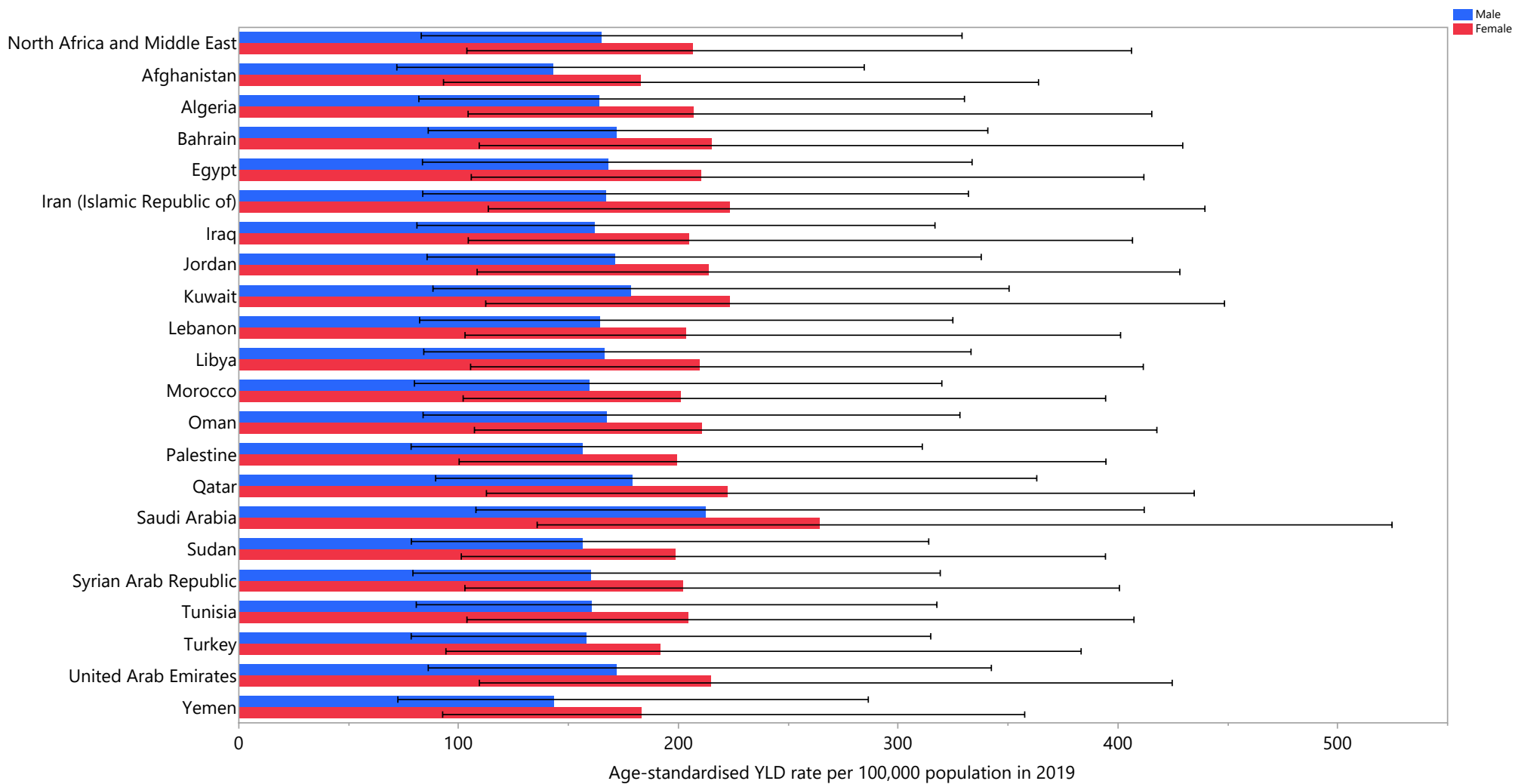

Supplement: Supplementary Figure S5 — Age-standardized YLDs rate of osteoarthritis (per 100,000 population) in the Middle East and North Africa region in 2019, by sex and country. YLD, years lived with disability (generated from data available from http://ghdx.healthdata.org/gbd-results-tool). [file Image_5.PDF]

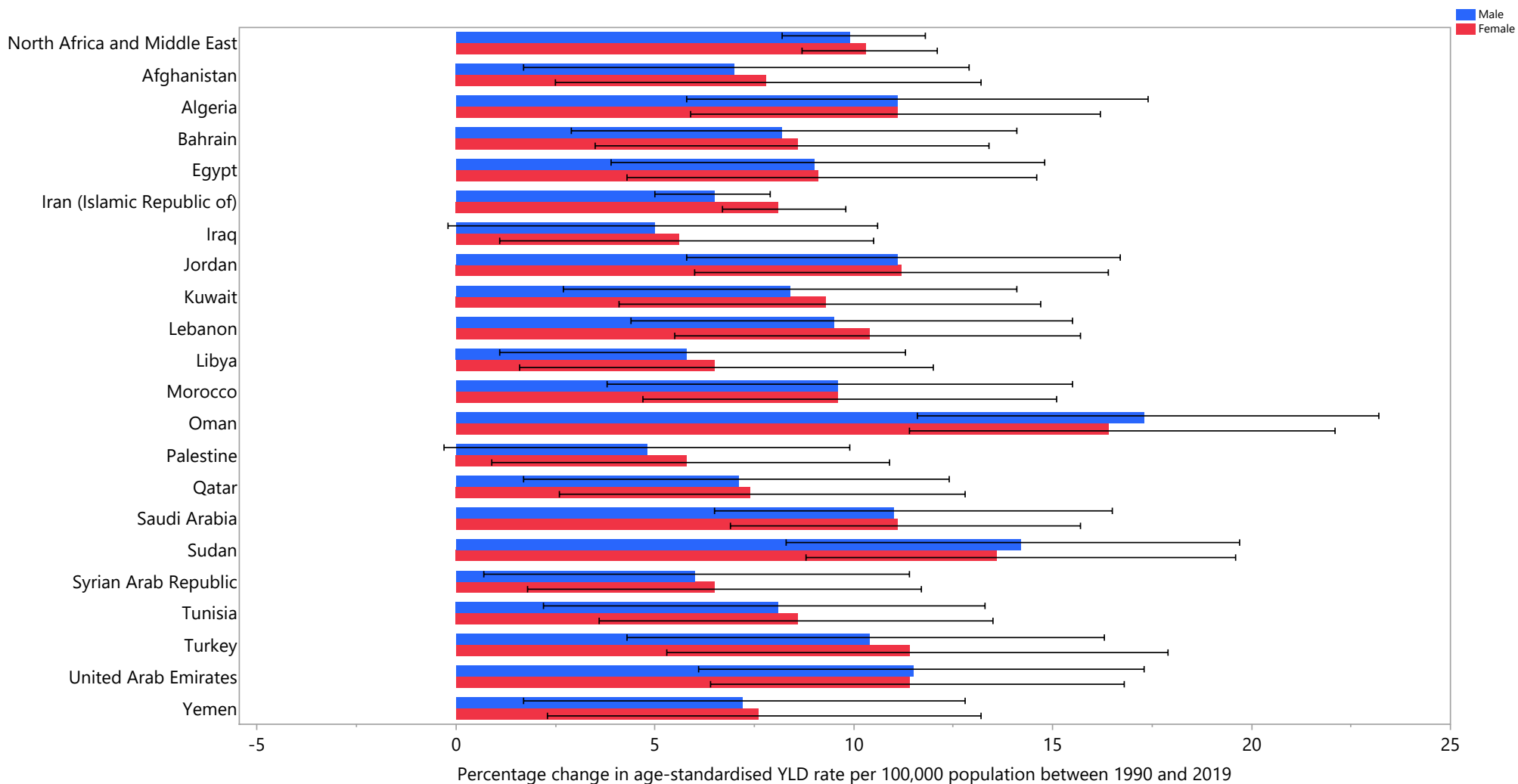

Supplement: Supplementary Figure S6 — The percentage change in the age-standardized YLDs of osteoarthritis in the Middle East and North Africa region from 1990 to 2019, by sex and country. YLD, years lived with disability (generated from data available from http://ghdx.healthdata.org/gbd-results-tool). [file Image_6.PDF]

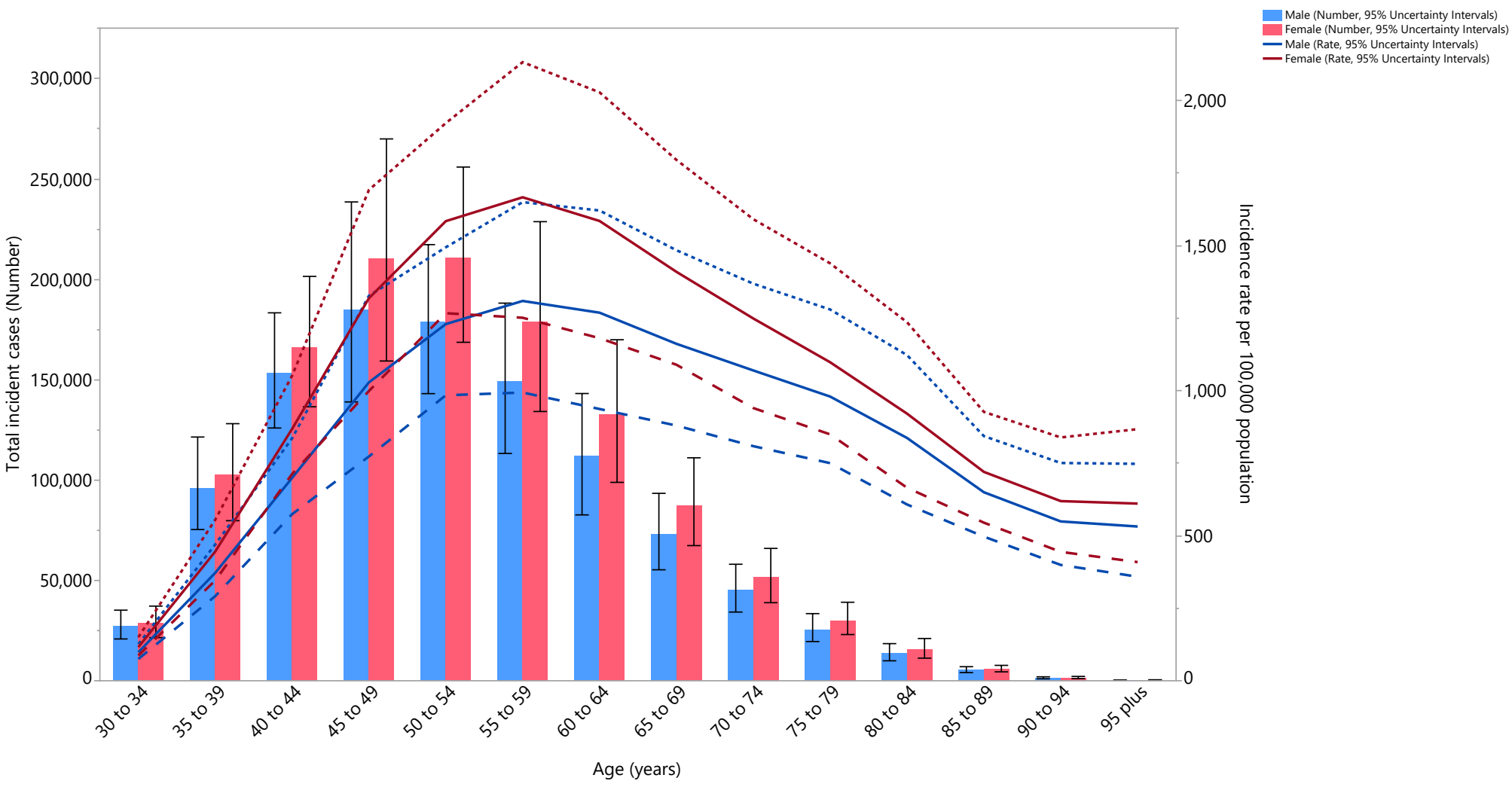

Supplement: Supplementary Figure S7 — Numbers of incident cases and incidence rate of osteoarthritis per 100,000 population in the Middle East and North Africa region, by age and sex in 2019; Dotted and dashed lines indicate 95% upper and lower uncertainty intervals, respectively (generated from data available from http://ghdx.healthdata.org/gbd-results-tool). [file Image_7.PDF]

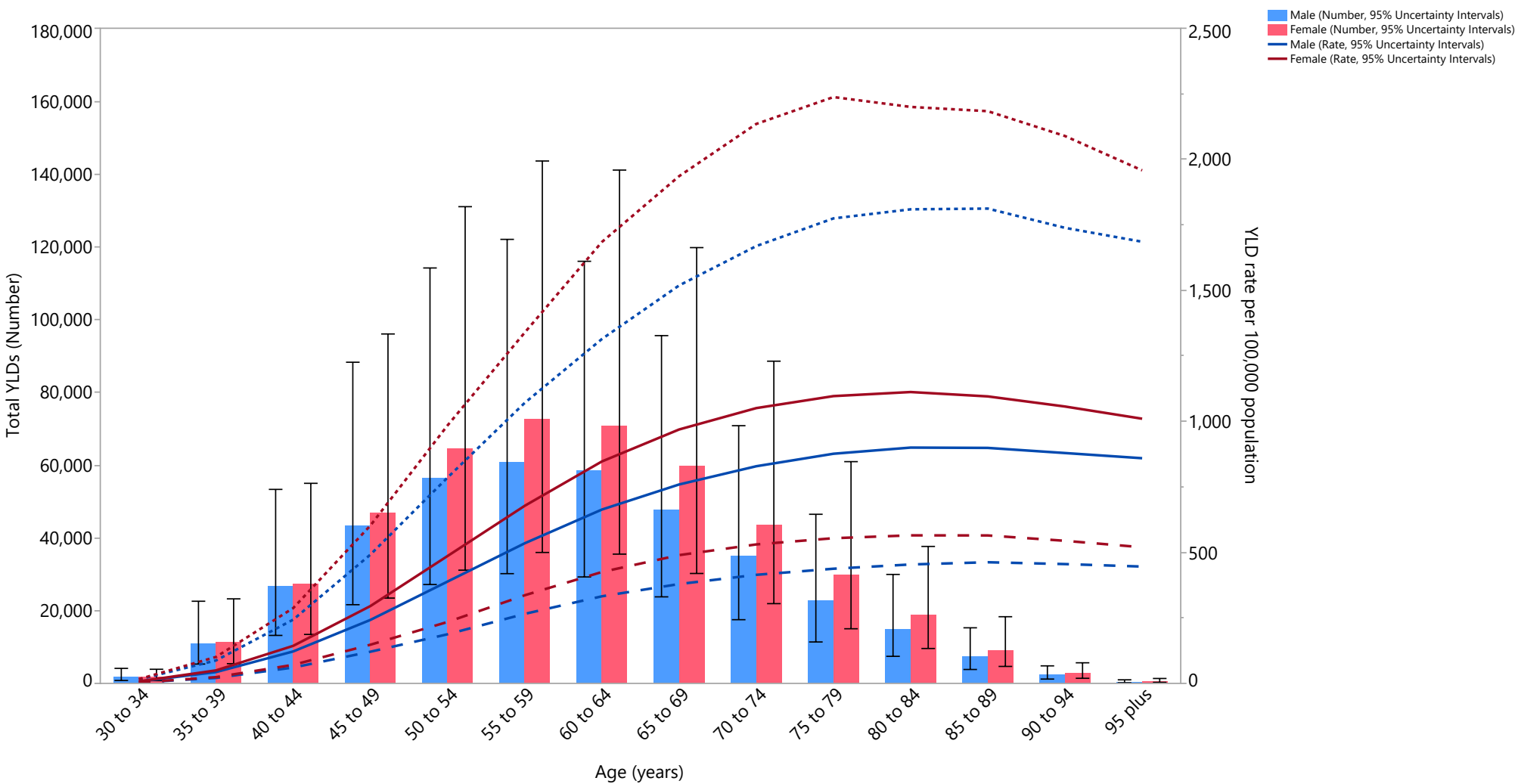

Supplement: Supplementary Figure S8 — Numbers of YLDs and YLD rate of osteoarthritis per 100,000 population in the Middle East and North Africa region, by age and sex in 2019; Dotted and dashed lines indicate 95% upper and lower uncertainty intervals, respectively. YLD, years lived with disability (generated from data available from http://ghdx.healthdata.org/gbd-results-tool). [file Image_8.PDF]

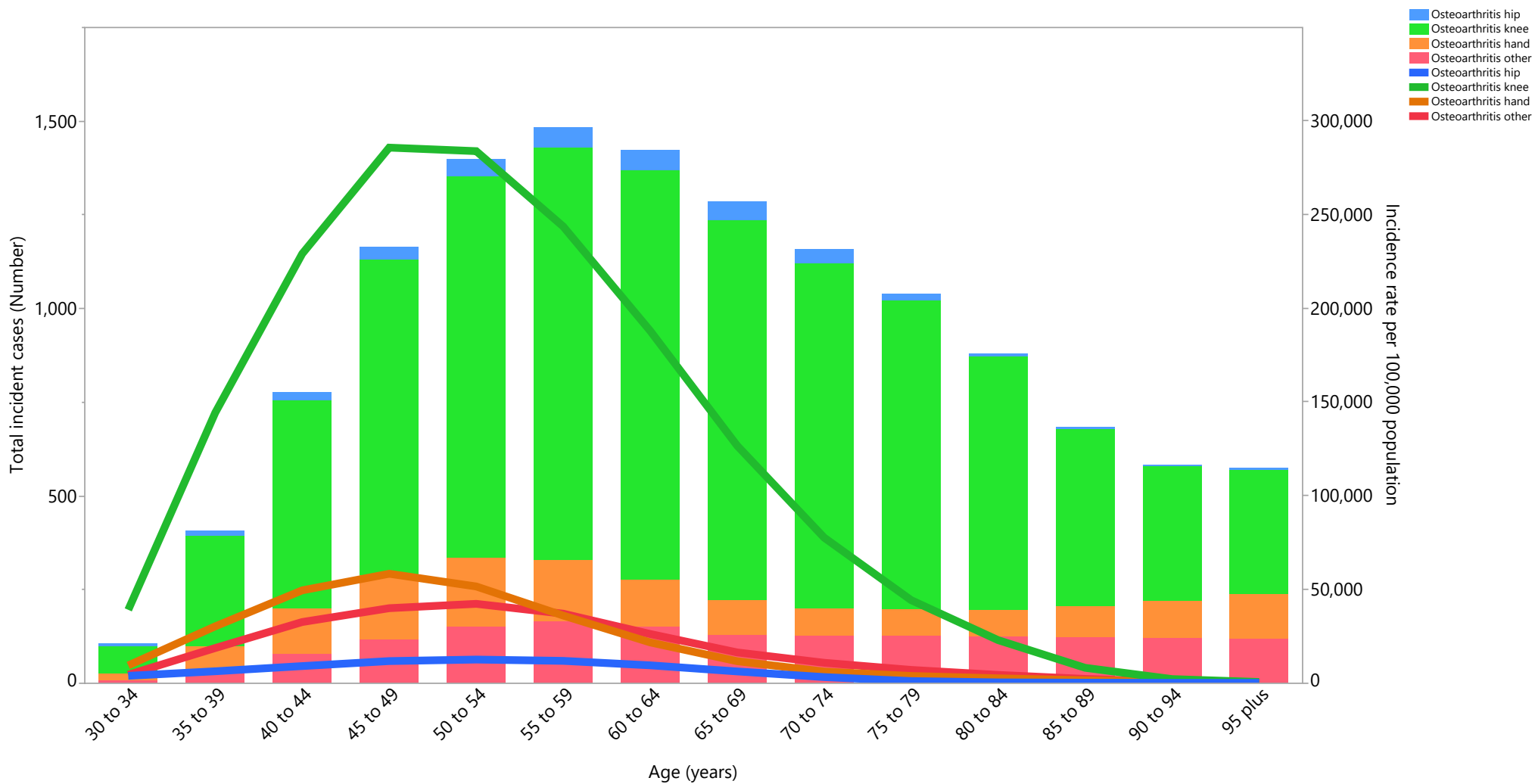

Supplement: Supplementary Figure S9 — Number of incident cases and incidence rate for osteoarthritis per 100,000 population in the Middle East and North Africa region, by age and cause in 2019 (generated from data available from http://ghdx.healthdata.org/gbd-results-tool). [file Image_9.PDF]

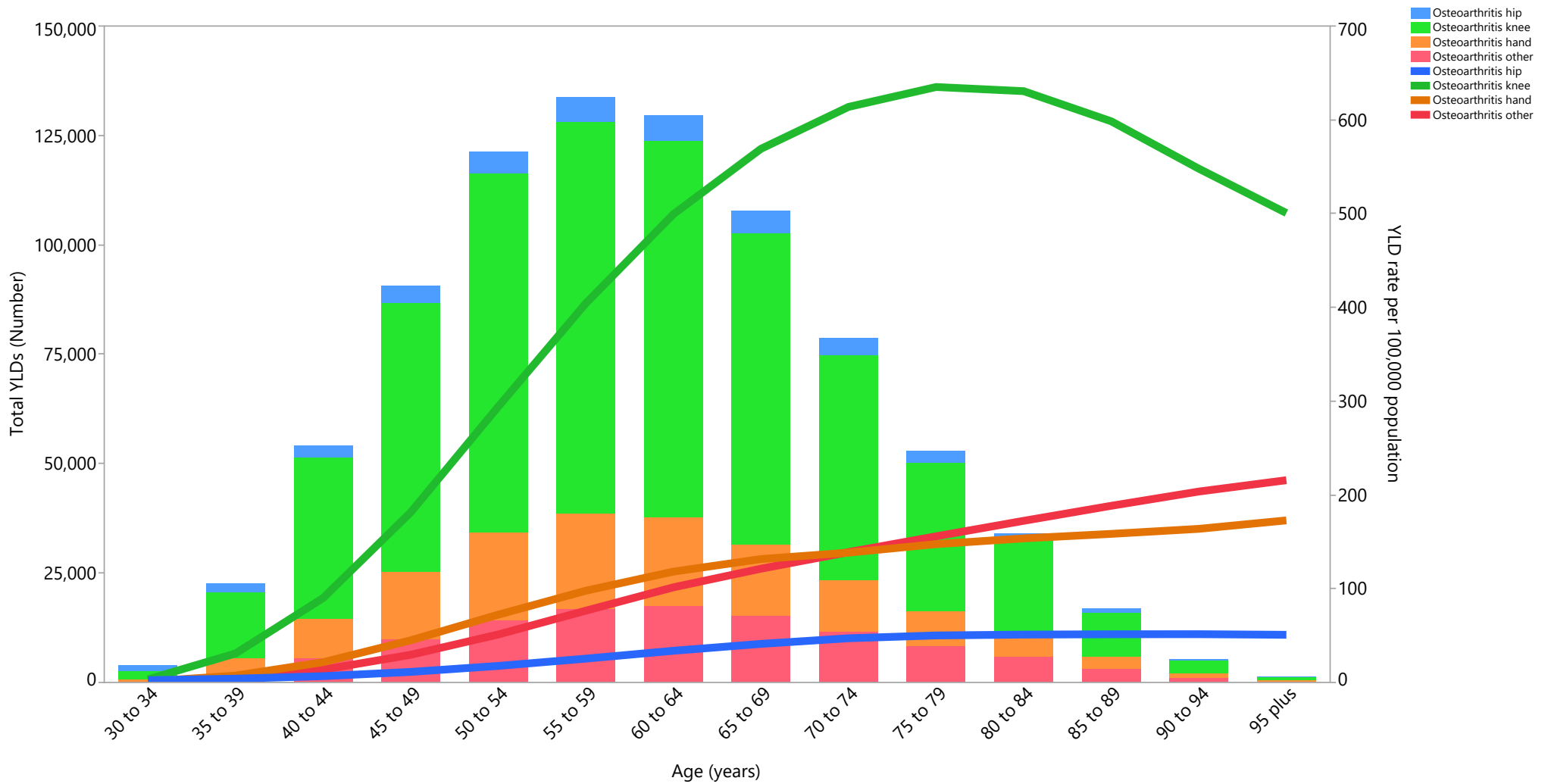

Supplement: Supplementary Figure S10 — Number of YLDs and YLD rate for osteoarthritis per 100,000 population in the Middle East and North Africa region, by age and cause in 2019. YLD= years lived with disability (generated from data available from http://ghdx.healthdata.org/gbd-results-tool). [file Image_10.PDF]
