# Supplementary material for: The Burden of Osteoarthritis in the Middle East and North Africa Region From 1990 to 2019
Source: Front Med (Lausanne). 2022 Jun 23;9:881391. doi: 10.3389/fmed.2022.881391 (PMC9261477; doi:10.3389/fmed.2022.881391)
Supplement: Supplementary Table S1 — Prevalence of osteoarthritis in 1990 and 2019 for both sexes and percentage change in age-standardized rates (ASRs) per 100,000 in the North Africa and the Middle East region (generated from data available from http://ghdx.healthdata.org/gbd-results-tool). [file Table_1.DOCX]

| **Table S1: Prevalence of osteoarthritis in 1990 and 2019 and the percentage change in the age-standardised rates (ASRs) per 100,000 in the North Africa and Middle East region**  **(Generated from data available from http://ghdx.healthdata.org/gbd-results-tool)** | | | | | |
| --- | --- | --- | --- | --- | --- |
|  | **1990** | | **2019** | | **Percentage change in ASRs per 100,000** |
|  | **No (95% UI)** | **ASRs per 100,000 (95% UI)** | **No (95% UI)** | **ASRs per 100,000 (95% UI)** |  |
| **North Africa and Middle East** | **8682345 (7793117 , 9681655)** | **4889.5 (4404.9 , 5422.3)** | **24604611 (22080960 , 27327135)** | **5342.8 (4815.9 , 5907.8)** | **9.3 (8.1 , 10.5)** |
| **Afghanistan** | **328021 (291179 , 367224)** | **4490.3 (4011.4 , 5009.7)** | **665279 (590218 , 749421)** | **4874.1 (4363.9 , 5431.5)** | **8.5 (5.5 , 11.9)** |
| **Algeria** | **608601 (541674 , 681068)** | **4832 (4325.8 , 5373.6)** | **1882093 (1681187 , 2089085)** | **5320.3 (4765.6 , 5873.4)** | **10.1 (7 , 13.4)** |
| **Bahrain** | **10515 (9398 , 11819)** | **5128.2 (4608.2 , 5685.3)** | **72697 (64518 , 81706)** | **5471.3 (4915 , 6032.6)** | **6.7 (3.8 , 10)** |
| **Egypt** | **1523479 (1367251 , 1701057)** | **4999.9 (4509.8 , 5545.7)** | **3656548 (3277333 , 4064564)** | **5374.1 (4844.3 , 5936.9)** | **7.5 (4.3 , 11.1)** |
| **Iran (Islamic Republic of)** | **1427709 (1276694 , 1606168)** | **5224.1 (4699.5 , 5821.4)** | **4282092 (3844615 , 4781751)** | **5588.2 (5041.2 , 6228.6)** | **7 (6.1 , 8)** |
| **Iraq** | **404714 (364419 , 450612)** | **5100.9 (4585.7 , 5661.4)** | **1308928 (1167311 , 1459554)** | **5321.5 (4762.8 , 5895.9)** | **4.3 (1.2 , 7.5)** |
| **Jordan** | **71881 (63881 , 80150)** | **4991.1 (4471.3 , 5519.1)** | **399351 (357424 , 447816)** | **5470.4 (4922.8 , 6035.8)** | **9.6 (6.5 , 13)** |
| **Kuwait** | **37097 (33067 , 41673)** | **5154.1 (4642.1 , 5693.9)** | **183772 (163298 , 206192)** | **5627.6 (5049.7 , 6251.9)** | **9.2 (6.1 , 12.6)** |
| **Lebanon** | **114224 (101908 , 127494)** | **4857.6 (4350 , 5393.2)** | **277812 (250318 , 307359)** | **5370.7 (4832.9 , 5940.6)** | **10.6 (7.4 , 13.9)** |
| **Libya** | **97851 (87451 , 108769)** | **5065.7 (4551.9 , 5610.5)** | **300298 (268933 , 337344)** | **5416.8 (4883.3 , 6035.7)** | **6.9 (3.7 , 10.2)** |
| **Morocco** | **669131 (598434 , 747035)** | **4775.1 (4289.8 , 5309.3)** | **1713120 (1533283 , 1916511)** | **5209.1 (4679.8 , 5786.7)** | **9.1 (5.9 , 12.4)** |
| **Oman** | **34126 (30598 , 38295)** | **4679 (4207.5 , 5211.6)** | **117159 (104084 , 131554)** | **5361.9 (4819 , 5945.5)** | **14.6 (11.3 , 18.1)** |
| **Palestine** | **43520 (38998 , 48601)** | **4962.2 (4442.6 , 5501.8)** | **133600 (120025 , 149034)** | **5191.1 (4676 , 5749.6)** | **4.6 (1.5 , 7.7)** |
| **Qatar** | **8503 (7482 , 9605)** | **5255.9 (4711.3 , 5834.9)** | **83742 (73719 , 94909)** | **5508 (4952.1 , 6115.1)** | **4.8 (1 , 8.1)** |
| **Saudi Arabia** | **388330 (344123 , 437303)** | **6017.8 (5383.3 , 6768.4)** | **1500332 (1324931 , 1700792)** | **6601.3 (5941.5 , 7358.3)** | **9.7 (7 , 12.5)** |
| **Sudan** | **427067 (380643 , 478365)** | **4518 (4024.5 , 5040.9)** | **1005517 (896511 , 1127167)** | **5081.9 (4555.5 , 5652.7)** | **12.5 (8.9 , 15.9)** |
| **Syrian Arab Republic** | **266254 (238016 , 297447)** | **4894.5 (4404.4 , 5440.3)** | **698433 (622281 , 780321)** | **5218.7 (4677.5 , 5794.9)** | **6.6 (3.4 , 9.9)** |
| **Tunisia** | **249953 (223550 , 279305)** | **4846.9 (4351.7 , 5387.7)** | **684103 (610552 , 759467)** | **5268.9 (4723.7 , 5828.4)** | **8.7 (5.6 , 11.7)** |
| **Turkey** | **1709749 (1517995 , 1909658)** | **4613.2 (4093.6 , 5121.4)** | **4606241 (4093835 , 5152806)** | **5089.5 (4532.3 , 5694.9)** | **10.3 (6.7 , 14.4)** |
| **United Arab Emirates** | **28991 (25529 , 33009)** | **4889 (4409.7 , 5440.2)** | **339146 (296726 , 386821)** | **5318.6 (4780.7 , 5879.2)** | **8.8 (5.2 , 12.4)** |
| **Yemen** | **226789 (203174 , 253509)** | **4477.7 (4030.7 , 4986.2)** | **669350 (599689 , 748587)** | **4770.5 (4293.7 , 5293.9)** | **6.5 (3.3 , 10)** |
