# Supplementary material for: The Burden of Osteoarthritis in the Middle East and North Africa Region From 1990 to 2019
Source: Front Med (Lausanne). 2022 Jun 23;9:881391. doi: 10.3389/fmed.2022.881391 (PMC9261477; doi:10.3389/fmed.2022.881391)
Supplement: Supplementary Table S2 — Incidence of osteoarthritis in 1990 and 2019 for both sexes and percentage change in age-standardized rates (ASRs) per 100,000 in the North Africa and the Middle East region (generated from data available from http://ghdx.healthdata.org/gbd-results-tool). [file Table_2.DOCX]

| **Table S2: Incidence of osteoarthritis in 1990 and 2019 and the percentage change in the age-standardised rates (ASRs) per 100,000 in the North Africa and Middle East region**  **(Generated from data available from http://ghdx.healthdata.org/gbd-results-tool)** | | | | | |
| --- | --- | --- | --- | --- | --- |
|  | **1990** | | **2019** | | **Percentage change in ASRs per 100,000** |
|  | **No (95% UI)** | **ASRs per 100,000 (95% UI)** | **No (95% UI)** | **ASRs per 100,000 (95% UI)** |  |
| **North Africa and Middle East** | **795083 (705541 , 895727)** | **393.5 (348.5 , 442.2)** | **2292214 (2035506 , 2570668)** | **430.4 (382.2 , 481.9)** | **9.4 (8.3 , 10.5)** |
| **Afghanistan** | **27914 (24526 , 31556)** | **362.9 (321.6 , 408.5)** | **69705 (60653 , 79550)** | **391.1 (346.1 , 441.8)** | **7.8 (4.7 , 11.5)** |
| **Algeria** | **53832 (47881 , 60751)** | **388.5 (345.7 , 438.8)** | **169449 (149048 , 190369)** | **427.5 (378 , 478.6)** | **10 (6.9 , 13.6)** |
| **Bahrain** | **1166 (1031 , 1315)** | **406.8 (361.3 , 454.2)** | **7927 (6928 , 8953)** | **434.4 (384.2 , 483.1)** | **6.8 (3.7 , 10.1)** |
| **Egypt** | **141417 (125013 , 160285)** | **400.7 (356.5 , 451.1)** | **340162 (300085 , 381427)** | **432.8 (383.2 , 484.5)** | **8 (4.4 , 11.5)** |
| **Iran (Islamic Republic of)** | **129719 (114779 , 146476)** | **414.6 (369.2 , 466.7)** | **385983 (342697 , 434999)** | **445 (395.9 , 501.9)** | **7.3 (6.4 , 8.4)** |
| **Iraq** | **36716 (32669 , 41227)** | **407.8 (362.9 , 458.2)** | **125093 (109828 , 141426)** | **425.8 (378.8 , 475.4)** | **4.4 (1.2 , 7.8)** |
| **Jordan** | **6989 (6157 , 7890)** | **399.5 (354.6 , 447.9)** | **38919 (34279 , 43848)** | **437.7 (389.5 , 489.4)** | **9.6 (6.1 , 13)** |
| **Kuwait** | **4227 (3723 , 4782)** | **410 (363.6 , 458.7)** | **20581 (18103 , 23375)** | **451.6 (400.4 , 505.7)** | **10.1 (6.7 , 13.9)** |
| **Lebanon** | **9812 (8638 , 11067)** | **388.7 (344.7 , 437.2)** | **22535 (19987 , 25317)** | **430 (381.5 , 483.4)** | **10.6 (7.3 , 14.2)** |
| **Libya** | **8916 (7857 , 10008)** | **404.3 (358.4 , 453.3)** | **29168 (25689 , 32984)** | **433.9 (385.1 , 488.1)** | **7.3 (3.7 , 10.5)** |
| **Morocco** | **60318 (53510 , 68156)** | **383.6 (340 , 431.7)** | **151245 (133506 , 171160)** | **418.4 (372.5 , 471.3)** | **9.1 (5.8 , 12.4)** |
| **Oman** | **3610 (3176 , 4066)** | **371.1 (329.3 , 417.6)** | **13756 (12170 , 15528)** | **424.6 (377.4 , 475.1)** | **14.4 (10.9 , 18.2)** |
| **Palestine** | **3807 (3365 , 4289)** | **398.2 (351.6 , 448)** | **12852 (11290 , 14494)** | **415.3 (370.3 , 465.4)** | **4.3 (1 , 7.6)** |
| **Qatar** | **1139 (995 , 1306)** | **414 (367.6 , 464)** | **10579 (9244 , 12009)** | **439.1 (390.5 , 489.9)** | **6.1 (2.3 , 10)** |
| **Saudi Arabia** | **38187 (33760 , 43725)** | **453.1 (402.3 , 508.9)** | **168749 (147967 , 193393)** | **504 (447.9 , 565.1)** | **11.2 (8 , 14.5)** |
| **Sudan** | **38887 (34375 , 43921)** | **364.1 (322.4 , 410.1)** | **97698 (86070 , 110443)** | **410.3 (363.9 , 462.8)** | **12.7 (9.1 , 16.4)** |
| **Syrian Arab Republic** | **24360 (21451 , 27375)** | **393.5 (350.1 , 441.5)** | **62346 (54723 , 70683)** | **420.5 (373.8 , 472.9)** | **6.8 (3.7 , 10.2)** |
| **Tunisia** | **21748 (19270 , 24517)** | **389.8 (346.4 , 438.1)** | **57298 (50683 , 64574)** | **422.8 (375.9 , 475.5)** | **8.5 (5 , 11.8)** |
| **Turkey** | **156223 (137193 , 175547)** | **379.8 (334.8 , 425)** | **393382 (347298 , 440721)** | **416.6 (369.7 , 466)** | **9.7 (5.9 , 13.4)** |
| **United Arab Emirates** | **3986 (3466 , 4580)** | **386.4 (343.6 , 432.4)** | **46556 (40342 , 53570)** | **423 (377 , 477.6)** | **9.5 (5.7 , 12.9)** |
| **Yemen** | **21574 (19032 , 24362)** | **357.6 (317.4 , 401.7)** | **65904 (58348 , 74424)** | **383.5 (340.9 , 430.6)** | **7.3 (3.9 , 10.5)** |
