# Supplementary material for: The Burden of Osteoarthritis in the Middle East and North Africa Region From 1990 to 2019
Source: Front Med (Lausanne). 2022 Jun 23;9:881391. doi: 10.3389/fmed.2022.881391 (PMC9261477; doi:10.3389/fmed.2022.881391)
Supplement: Supplementary Table S3 — YLDs due to osteoarthritis in 1990 and 2019 for both sexes and percentage change in age-standardized rates (ASRs) per 100,000 in the North Africa and the Middle East region (generated from data available from http://ghdx.healthdata.org/gbd-results-tool). [file Table_3.DOCX]

| **Table S3: YLDs due to osteoarthritis in 1990 and 2019 and the percentage change in the age-standardised rates (ASRs) per 100,000 in the North Africa and Middle East region**  **(Generated from data available from http://ghdx.healthdata.org/gbd-results-tool)** | | | | | |
| --- | --- | --- | --- | --- | --- |
|  | **1990** | | **2019** | | **Percentage change in ASRs per 100,000** |
|  | **No (95% UI)** | **ASRs per 100,000 (95% UI)** | **No (95% UI)** | **ASRs per 100,000 (95% UI)** |  |
| **North Africa and Middle East** | **299619 (150145 , 599218)** | **168.6 (84.4 , 336.8)** | **852891 (425290 , 1687138)** | **185.4 (92.8 , 370.2)** | **10 (8.7 , 11.4)** |
| **Afghanistan** | **11055 (5618 , 22245)** | **151 (76.4 , 304.6)** | **22313 (11329 , 44226)** | **163.9 (83.1 , 326.4)** | **8.5 (4.9 , 12.4)** |
| **Algeria** | **21093 (10669 , 42573)** | **167.1 (84.5 , 334.3)** | **65452 (32760 , 129991)** | **185.1 (92.9 , 369.4)** | **10.8 (6.9 , 14.5)** |
| **Bahrain** | **363 (182 , 732)** | **176.7 (89.4 , 350.3)** | **2505 (1248 , 4957)** | **188.7 (95 , 375.5)** | **6.8 (3.3 , 10.8)** |
| **Egypt** | **52838 (26831 , 105851)** | **173.5 (87.8 , 345.6)** | **127344 (62958 , 254833)** | **187.2 (93.6 , 377.1)** | **7.9 (4.3 , 12.3)** |
| **Iran (Islamic Republic of)** | **49531 (25023 , 97062)** | **181 (91.8 , 355)** | **149354 (75490 , 293270)** | **195.3 (98.7 , 381)** | **7.9 (6.9 , 9.1)** |
| **Iraq** | **13850 (6937 , 27653)** | **174.5 (87.4 , 349.2)** | **45113 (22709 , 89017)** | **183.8 (92.7 , 367.3)** | **5.4 (1.7 , 9.3)** |
| **Jordan** | **2485 (1258 , 4893)** | **172.8 (87.9 , 343.1)** | **13920 (6964 , 27536)** | **191.3 (95.2 , 383.9)** | **10.7 (7.1 , 14.5)** |
| **Kuwait** | **1287 (641 , 2582)** | **179.6 (90.6 , 361.3)** | **6374 (3165 , 12712)** | **196.7 (98.5 , 392)** | **9.5 (5.7 , 13.7)** |
| **Lebanon** | **3938 (1986 , 7903)** | **167.3 (84.6 , 335)** | **9609 (4871 , 18962)** | **185.7 (94.2 , 366.5)** | **11 (7.4 , 14.9)** |
| **Libya** | **3391 (1708 , 6691)** | **175.7 (88.8 , 349.7)** | **10363 (5240 , 20678)** | **187.6 (94.4 , 374.5)** | **6.8 (3.1 , 10.8)** |
| **Morocco** | **23100 (11654 , 46350)** | **164.8 (83.5 , 327)** | **59341 (29857 , 117164)** | **180.5 (90.4 , 357.2)** | **9.5 (5.7 , 13.5)** |
| **Oman** | **1171 (585 , 2300)** | **160.3 (80.9 , 314.1)** | **4051 (2027 , 8146)** | **185.8 (93 , 364.3)** | **15.9 (11.9 , 20.1)** |
| **Palestine** | **1500 (744 , 2979)** | **170.9 (85.2 , 340.1)** | **4590 (2321 , 9133)** | **178.7 (89.5 , 350.3)** | **4.5 (1 , 8.4)** |
| **Qatar** | **292 (143 , 585)** | **181.4 (91 , 358.2)** | **2878 (1436 , 5806)** | **189.5 (95.4 , 379.6)** | **4.4 (0.3 , 8.2)** |
| **Saudi Arabia** | **13566 (6835 , 27074)** | **210.7 (107 , 415)** | **52545 (26610 , 104734)** | **233.1 (118.8 , 457.8)** | **10.7 (7.3 , 13.8)** |
| **Sudan** | **14667 (7373 , 29380)** | **155 (78.4 , 308.5)** | **34719 (17492 , 68873)** | **175.8 (88.9 , 351.2)** | **13.4 (9.4 , 17.6)** |
| **Syrian Arab Republic** | **9214 (4631 , 18360)** | **169.4 (84.5 , 340.9)** | **24154 (12077 , 47650)** | **180.4 (90.7 , 357.1)** | **6.5 (2.7 , 10.1)** |
| **Tunisia** | **8674 (4367 , 17216)** | **167.9 (84.2 , 332.4)** | **23773 (12000 , 47643)** | **183 (92.6 , 368.2)** | **9 (5.1 , 12.7)** |
| **Turkey** | **58654 (28674 , 117794)** | **158.2 (78.4 , 317)** | **159045 (78292 , 319394)** | **175.7 (86.7 , 352.9)** | **11.1 (6.9 , 15.9)** |
| **United Arab Emirates** | **995 (494 , 2011)** | **168.2 (85.3 , 331.7)** | **11637 (5755 , 23432)** | **183.5 (92.6 , 362.8)** | **9.1 (5 , 13.1)** |
| **Yemen** | **7752 (3926 , 15317)** | **153 (77.1 , 300.7)** | **22943 (11442 , 45262)** | **163.7 (82.1 , 326)** | **7 (3.3 , 11)** |
